# Enhancer of polycomb coordinates multiple signaling pathways to promote both cyst and germline stem cell differentiation in the *Drosophila* adult testis

**DOI:** 10.1371/journal.pgen.1006571

**Published:** 2017-02-14

**Authors:** Lijuan Feng, Zhen Shi, Xin Chen

**Affiliations:** Department of Biology, The Johns Hopkins University, Baltimore, MD, United States of America; Stanford University School of Medicine, UNITED STATES

## Abstract

Stem cells reside in a particular microenvironment known as a niche. The interaction between extrinsic cues originating from the niche and intrinsic factors in stem cells determines their identity and activity. Maintenance of stem cell identity and stem cell self-renewal are known to be controlled by chromatin factors. Herein, we use the *Drosophila* adult testis which has two adult stem cell lineages, the germline stem cell (GSC) lineage and the cyst stem cell (CySC) lineage, to study how chromatin factors regulate stem cell differentiation. We find that the chromatin factor Enhancer of Polycomb [E(Pc)] acts in the CySC lineage to negatively control transcription of genes associated with multiple signaling pathways, including JAK-STAT and EGF, to promote cellular differentiation in the CySC lineage. E(Pc) also has a non-cell-autonomous role in regulating GSC lineage differentiation. When E(Pc) is specifically inactivated in the CySC lineage, defects occur in both germ cell differentiation and maintenance of germline identity. Furthermore, compromising Tip60 histone acetyltransferase activity in the CySC lineage recapitulates loss-of-function phenotypes of E(Pc), suggesting that Tip60 and E(Pc) act together, consistent with published biochemical data. In summary, our results demonstrate that E(Pc) plays a central role in coordinating differentiation between the two adult stem cell lineages in *Drosophila* testes.

## Author summary

Tissue maintenance and repair rely on adult stem cells, which can divide to generate new stem cells as well as cells committed for becoming specific cell types. Stem cell activity needs to be tightly controlled because insufficient or unlimited stem cell division may lead to tissue degeneration or tumorigenesis. This control depends not only on stem cells themselves, but also on the microenvironment where stem cells reside. The chromatin structure of stem cells is crucial to determine their activities. The signaling pathways connecting stem cells with their microenvironment is also important. Here we ask how chromatin factors interact with signaling pathways in determining stem cell activity. We use *Drosophila* adult testis as a model system, in which two types of stem cells co-exist and interact: germline stem cells and somatic stem cells. We find that a chromatin regulator called Enhancer of Polycomb [E(Pc)] acts in somatic cells to promote germ cell differentiation and maintain germ cell fate. This regulation is mediated by several signaling pathways, such as EGF and JAK-STAT pathways. E(Pc) also works with another chromatin regulator, the histone acetyltransferase Tip60, in somatic cells. Insufficient activity of the E(Pc) homolog in human leads to cancers. Our studies of E(Pc) may help understanding its roles as a tumor suppressor.

## Introduction

In physiological condition, adult stem cells are surrounded by other cell types and extracellular matrix. Recent studies have demonstrated a dynamic crosstalk between stem cells and their surrounding microenvironment termed as the stem cell niche [1]. Signaling molecules emanating from this niche contribute to the balance between self-renewal and differentiation of adult stem cells, which is essential for the maintenance of tissue homeostasis and regeneration in response to injury. Therefore a fundamental question in stem cell biology is how extrinsic cues and intrinsic factors cooperate to determine and maintain stem cell identity and activity.

**Fig 1:**
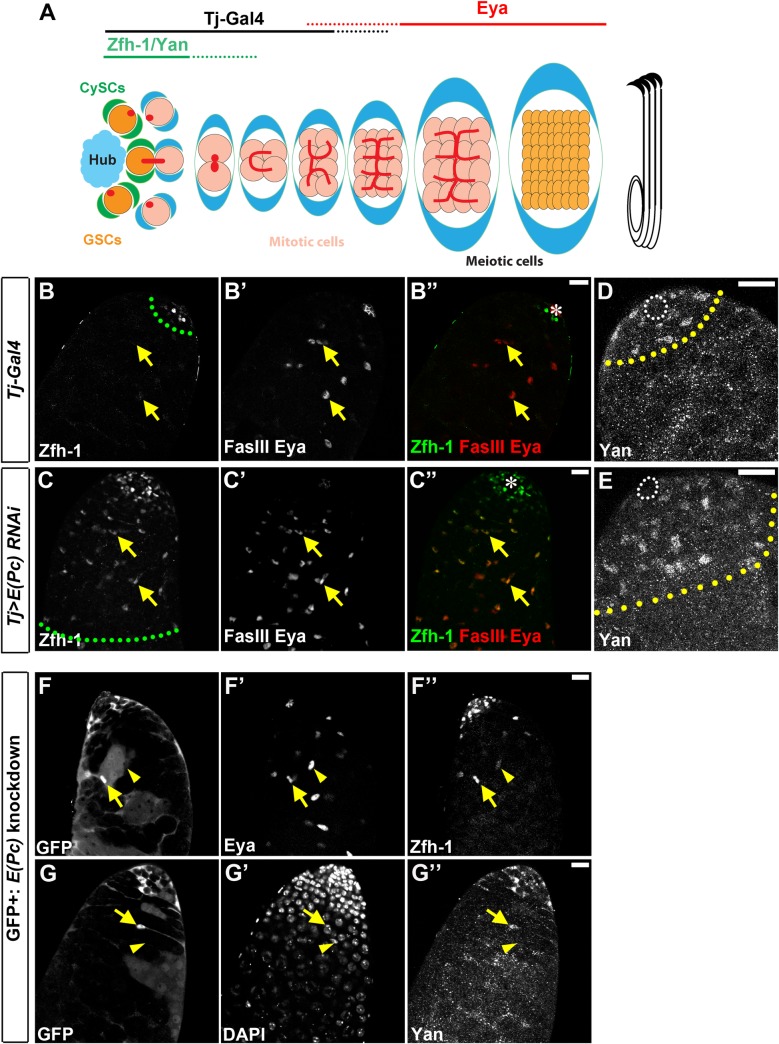
E(Pc) downregulates CySC-enriched transcription factors. **(A)** A schematic diagram of *Drosophila* adult testes. CySCs: cyst stem cells; GSCs: germline stem cells. **(B-B”)** In *Tj-Gal4* control testes, Zfh-1-positive early cyst cell zone (green dashed line) is separable spatially from the region with later stage cyst cells labeled by Eya (yellow arrows). **(C-C”)** In *Tj>E(Pc) RNAi* testes, Zfh-1-positive cell zone expands (green dashed line) with some cells co-expressing Eya (yellow arrows). **(D)** In *Tj-Gal4* control testes, another marker Yan is highly enriched in CySCs and early cyst cells (yellow dashed line), a few cell-diameter away from the hub region (white outline). **(E)** In *Tj>E(Pc) RNAi* testes, Yan-positive cell zone (yellow dashed line) expands and is further away from hub (white outline). **(F-G”)** GFP-positive cells represent cells with *E(Pc)* knockdown. Compared with the GFP-negative and Eya-positive cyst cell (yellow arrowhead in **F-F”**), the neighboring GFP-positive and Eya-positive cyst cell (yellow arrow in **F-F”**) has higher Zfh-1 signal. Similarly, compared with GFP-negative and DAPI-positive cyst cell (yellow arrowhead in **G-G”**), the neighboring GFP-positive and DAPI-positive cyst cell (yellow arrow in **G-G”**) has higher Yan signal. Asterisk: hub. Scale bar: 20μm. See also S1 Fig.

Two adult stem cell lineages reside in the *Drosophila* adult testis: the germline stem cell (GSC) lineage and the cyst stem cell (CySC) lineage (Fig 1A). Both GSCs and CySCs attach to a group of post-mitotic somatic cells called hub cells and serve as a niche for each other [2]. Both GSCs and CySCs undergo asymmetric cell divisions to produce one self-renewed stem cell and one differentiated daughter cell in each lineage [3,4]. The differentiated daughter cell in the GSC lineage is called a gonialblast (GB), which subsequently undergoes a transit-amplifying stage with exactly four rounds of mitosis. After exiting the mitotic expansion, germ cells enter the meiotic stage with an elongated G2 phase as spermatocytes, in which a robust gene expression program is initiated to prepare them for meiotic divisions and spermatid differentiation. On the other hand, the differentiated daughter cell in the CySC lineage becomes a cyst cell, which never divides again. Two cyst cells encapsulate synchronously dividing and differentiating germ cells and form a distinct germ cell cyst. Ectopic niche formation may result in an expanded stem cell population and lead to tumor formation [5]. Conversely, dysfunction of stem cells from an impaired niche is associated with compromised injury recovery, degenerative disease and aging [6]. Studies using *Drosophila* gonads have improved our understanding of the regulatory mechanisms within the stem cell niche [2,7].

*Drosophila* testis has provided an excellent model system by which to study the crosstalk among different stem cell lineages. For example, it has been shown that the JAK-STAT and TGF-β signaling pathways are important for male GSC maintenance through interactions with CySCs [8,9,10,11]. The JAK-STAT signaling pathway ligand Unpaired (Upd) is secreted by hub cells to activate the downstream transcription factor Stat92E in both CySCs and GSCs for their maintenance [8,9,11,12,13,14,15]. In addition, the EGF signaling pathway has been shown to control cyst cells to encapsulate germ cells and allow for their proper differentiation [16,17,18,19,20]. A protease called Stet acts in germ cells to cleave the Spitz (Spi) ligand to stimulate EGF signaling in cyst cells [18]. Activation of EGF signaling ensures encapsulation of germ cells by the cyst cell and promotes germ cell differentiation [16,17,18,19,20,21,22,23,24].

Most studies on germline and soma communication have focused on signaling pathways, while most work on chromatin regulators mainly addressed their cell-autonomous functions. However, recent studies have demonstrated their cooperation (reviewed by [25,26]). For example, JAK-STAT signaling in both GSCs and CySCs is positively regulated by the nucleosome remodeling factor (NURF) [27]. On the other hand, the *Socs36E* gene encodes an inhibitor of the JAK-STAT signaling pathway, which is critical for maintaining balance between GSCs and CySCs at the niche [12,15,23,24]. Our previous studies showed that an H3K27me3-specific histone demethylase, dUTX, acts upstream of and negatively regulates the JAK-STAT signaling pathway through maintaining active *Socs36E* transcription [28]. Moreover, genes of the EGF signaling pathway might be directly regulated by the H3K27me3 methyltransferase Enhancer of Zeste [E(z)] in cyst cells to promote germ cell differentiation [29]. However, identification of more crosstalk between signaling pathways and chromatin factors in the CySC lineage has been hampered by the limited number of cyst cells for experimental methods, such as Chromatin immunoprecipitation (ChIP) and protein co-immunoprecipitation (Co-IP). Thus, regulation of CySC differentiation and the coordination of CySC differentiation with neighboring germ cells remain to be fully addressed.

The enhancer of Polycomb [E(Pc)] gene is known as a putative Polycomb group (PcG) gene which is conserved from yeast to mammals, suggesting its crucial roles in regulating chromatin structure across species. The yeast homolog of E(Pc) was identified as a component of the NuA4 (nucleosome acetyltransferase of H4) histone acetyltransferase (HAT) complex [30,31,32], which has been shown to contribute to the hyperacetylation state of both H4 and H2A to stimulate transcription [33,34,35,36,37]. Abnormal activity of the human E(Pc) homolog called EPC1 has been shown to cause T-cell leukemia/lymphoma [38]. However, the molecular and cellular mechanisms of *in vivo* functions of E(Pc) have been elusive.

Here we use the *Drosophila* adult testis as a model system to study functions of E(Pc) in endogenous adult stem cell lineages. We find that E(Pc) promotes cyst cell differentiation by downregulating CySC-enriched transcription factors Zfh-1 and Yan. Loss of such repression by E(Pc) in CySC lineage blocks both cyst and germ cell differentiation, which causes both CySC-like and early-stage germline tumors, including GSC-like tumor and spermatogonial tumor. In addition, when E(Pc) is specifically knocked down in CySC lineage, some germ cells ectopically turn on expression of cyst cell markers such as Zfh-1 and Yan. When ChIP followed by high-throughput sequencing (ChIP-seq) is performed specifically in cyst cells, many components of key signaling pathways are identified as direct targets of E(Pc), suggesting its central role in coordinating the crosstalk between CySC and GSC lineages. Finally, inactivation of Tip60 HAT activity in the CySC lineage leads to defects resembling loss-of-function phenotypes of E(Pc), suggesting that they act together *in vivo*. Thus, E(Pc) establishes proper chromatin state in cyst cells to provide an instructive microenvironment to guide germ cell differentiation and protect germline identity.

## Results

### E(Pc) downregulates CySC-enriched transcription factors Zfh-1 and Yan

To understand the *in vivo* functions of E(Pc) in the *Drosophila* adult testis, we first characterized the E(Pc) expression pattern. Using a GFP-tagged genomic rescue transgene (Materials and Methods), the nuclear E(Pc) gDNA-GFP signal was detected in both germ cells and cyst cells throughout the adult testis (S1A–S1A” and S1B–S1B” Fig). The nuclear localization of E(Pc) is consistent with the prediction that E(Pc) is a chromatin regulator [39].

Because E(Pc) is required for early development and the null allele is lethal at embryonic or early larval stage [40], we first studied the functions of E(Pc) in adult testes using the RNAi knockdown strategy [41]. When E(Pc) was knocked down in cyst cells using a cyst cell-specific Tj-Gal4 driver [42] paired with two independent RNAi lines [E(Pc) dsRNA or E(Pc) shRNA, when phenotypes from these two lines are indistinguishable we called them E(Pc) RNAi, see Materials and Methods], the GFP signal representing the E(Pc) gDNA-GFP fusion protein level was greatly reduced in cyst cells compared to the neighboring germ cells (S1C–S1C” Fig), suggesting efficient knockdown.

In the CySC lineage, two transcription factors are known to express in a spatiotemporally specific manner. The first is zinc-finger homeodomain protein 1 (Zfh-1), a transcription repressor with multiple zinc finger domains and a homeodomain. It is highly expressed in CySCs and early cyst cells, and it is required for CySC maintenance [10]. The second is Eyes absent (Eya), which is expressed in later stage cyst cells. It is required for cyst cell differentiation [43]. Immunostaining experiments showed very few cyst cells (6.9±2.5) with overlapping Zfh-1 and Eya signals in the control testis (N = 44) (Fig 1B–1B”, S2A Fig). On the other hand, the percentage of testes with cyst cells carrying both Zfh-1 and Eya immunostaining signals was significantly increased in both *Tj>E(Pc) dsRNA* (N = 32) and *Tj>E(Pc) shRNA* (N = 35) testes (S2A Fig), most likely the result of overpopulation of CySC-like Zfh-1-expressing cells (Fig 1C–1C”). It has been shown that Zfh-1 overexpression in CySC lineage leads to overpopulation of both CySCs and GSCs [10]. Based on microarray analysis [44] and RNA-seq data [45], a transcription repressor, anterior open, often termed as Yan, is highly expressed in stem cell-enriched samples. Yan is an ETS domain-containing transcriptional repressor antagonizing the EGF signaling pathway [46], and it inhibits cellular differentiation [47]. Immunostaining experiments showed enriched Yan protein in CySCs and possibly their immediate daughter cells in the control testes (N = 22) (Fig 1D). By way of contrast, the number of Yan-positive cells increased in 72% of *Tj>E(Pc) dsRNA* (N = 18) and 74% of *Tj>E(Pc) shRNA* (N = 35) testes, (Fig 1E). Furthermore, immunostaining against the pan cyst cell marker Traffic jam (Tj) [48] and the later stage cyst cell marker Eya [43] both showed significantly increased Tj-positive and Eya-positive cells in *Tj>E(Pc) shRNA* testes (N = 31) (S2B–S2D Fig). These data suggest two major phenotypes in the CySC lineage upon knocking down E(Pc): first, the normal spatiotemporally specific expression pattern of CySC-lineage markers was not preserved. Second, there were excess cyst cells including both CySC-like cells and later stage cyst cells.

In addition to knock down *E(Pc)* in the entire CySC lineage, the *hs-FLP; Actin-FRT-stop-FRT-Gal4*, *UAS-GFP; UAS- E(Pc) shRNA* fly strain (Materials and Methods) was used to induce *E(Pc)* knockdown in a subset of cells in CySC lineage. When GFP-positive cells (arrows in Fig 1F–1F” and 1G–1G”) were compared with neighboring GFP-negative wild-type cells (arrowheads in Fig 1F–1F” and 1G–1G”) in the same testis under the same experimental condition, ectopic expression of Zfh-1 (arrow in Fig 1F”, N = 13 cells) and Yan (arrow in Fig 1G”, N = 11 cells) was detected exclusively in GFP-positive cells, consistent with the entire CySC lineage knockdown phenotype.

Because all E(Pc) knockdown experiments primarily used *Tj-Gal4* driver, histone H3-GFP was used as a reporter in both *Tj-Gal4>UAS-H3-GFP* (N = 45) and *Tj-Gal4>UAS-H3-GFP*, *UAS-E(Pc) shRNA* (N = 44) testes (S2E–S2F”’ Fig). Although GFP-positive cells increased in *E(Pc)* knockdown testes (S2E and S2F Fig), consistent with the overall increase of Tj-positive cells (S2D Fig), GFP signal was detected exclusively in the CySC lineage with no overlap with Vasa-positive germ cells (S2E”’ and S2F”’ Fig), suggesting that the cell type specificity of the *Tj-Gal4* driver is unaffected.

### E(Pc) is required in cyst cells to promote germ cell differentiation and maintain germline identity

**Fig 2:**
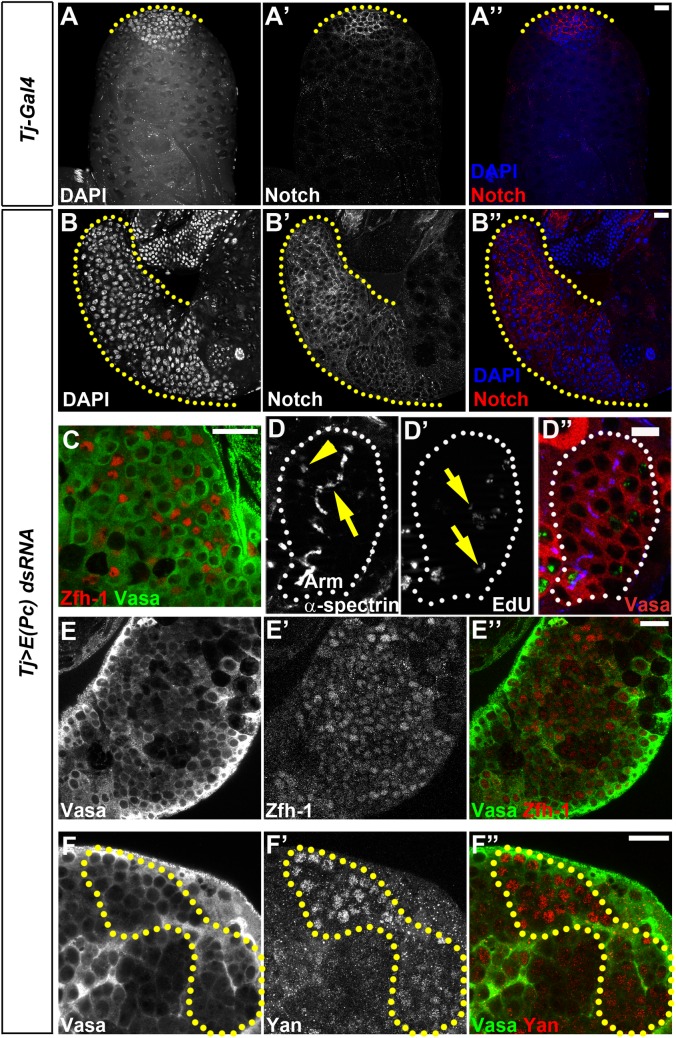
E(Pc) acts in cyst cells to promote germ cell differentiation and maintain germline identity. **(A-A”)** In *Tj-Gal4* control testes, DAPI bright region (yellow dashed line in **A**), and Notch positive cells (yellow dashed line in **A’**) represent GSCs and early-stage germ cells. **(B-B”)** Elongated DAPI bright region (yellow dashed line in **B**) and Notch-positive cell zone (yellow dashed line in **B’**) in *Tj>E(Pc) dsRNA* testes. (**C**) Immunostaining with germ cell marker Vasa (Green) and early cyst cell marker Zfh-1 in *Tj>E(Pc) dsRNA* testes: GSC- and GB-like germ cells intermingle with Zfh-1 positive cells. (**D-D”**) Immunostaining with antibodies against Armadillo and α–spectrin **(D)** in *Tj>E(Pc) dsRNA* testes show spermatogonial tumor cells interconnected with both round spectrosome (yellow arrowhead) and branched fusome (yellow arrow). EdU labeling in *Tj>E(Pc) dsRNA* testes (**D’**) show only a subset of overproliferating germ cells within one cyst are EdU-positive (yellow arrow). Scale bar: 10μm. (**E-F”**) In *Tj>E(Pc) dsRNA* testes, Vasa-positive germ cells (**E**, **F**, green in **E”** and **F”**) are also labeled with CySCs-enriched marker Zfh-1 (**E’**, red in **E”**) and Yan (**F’**, red in **F”**). Scale bar: 20μm. See also S2, S3, S4, S5, S6, S7 and S8 Figs.

The CySC lineage has been thought to play a supportive role for germ cell differentiation by enclosing germ cells and providing instructive signals for germline differentiation and survival [49,50,51]. We found that knockdown of *E(Pc)* in cyst cells using Tj-Gal4 led to excess early-stage germ cells in 43% of *Tj>E(Pc) dsRNA* testes (N = 40). Further reduction of E(Pc) levels, using a loss-of-function mutant *E(Pc)*^*1*^ [52] as heterozygotes, significantly enhanced the excess early germ cell phenotype to 70% of *Tj>E(Pc) dsRNA* testes (N = 20). Early stage germ cells visualized by bright DAPI staining [10,17] were restricted to the apical tip region in the control testes (Fig 2A and 2A”), but became expanded in the *E(Pc)* knockdown testes (Fig 2B and 2B”). Another early-stage germ cell marker, Notch [16], showed a confined immunostaining signal in the control testes (Fig 2A’ and 2A”), but significantly increased signal in the *E(Pc)* knockdown testes (Fig 2B’ and 2B”). The expansion of Notch-positive cells with DAPI bright nuclei is often associated with germline defects in the mitosis-to-meiosis transition, as shown previously [10,16,17].

We further analyzed the cellular properties of the excess germ cells in both *Tj>E(Pc) dsRNA* (Fig 2) and *Tj>E(Pc) shRNA* (S3 Fig) testes. In 12.5% of *Tj>E(Pc) dsRNA* testes (N = 40, Fig 2C) and 30% of *Tj>E(Pc) shRNA* testes (N = 37, S3A Fig), excess germ cells resembled GSC- or GB-tumor with round spectrosome structure intermingled with Zfh-1-positive CySC-like cells. In 30% of *Tj>E(Pc) dsRNA* testes (N = 40, white dotted outline in Fig 2D–2D”) and 40% of *Tj>E(Pc) shRNA* testes (N = 37, S3B and S3B’ Fig), excess germ cells were more like spermatogonial tumors with more than 16 Vasa-positive cells within one cyst, as visualized by anti-Armadillo staining delineating the encapsulating cyst cells (Fig 2D, S3B Fig). However, different from the continuous fusome structure in spermatogonial cysts in wild-type or control testes [53,54,55], germ cells within one cyst showed both dotted spectrosome (yellow arrowheads in Fig 2D, S3B and S3B’ Fig) and branched fusome (yellow arrows in Fig 2D, S3B and S3B’ Fig) structures, suggesting that these cells were not undergoing cell cycle in synchrony. This asynchrony was further confirmed when EdU (5-ethynyl-2′-deoxyuridine) incorporation assay was performed to label S-phase cells [56]. Only a subset of excess germ cells was labeled by EdU (arrows in Fig 2D’) in 54% of single cysts (N = 26) in *E(Pc)* knockdown testes.

Bag-of-marbles (Bam) is an important differentiation factor detectable in 4- to 16-cell spermatogonia in wild-type [57,58] and control testes (S4A and S4A’ Fig). In all testes with spermatogonial tumor (45% of *Tj>E(Pc) dsRNA* testes, N = 20; 57% of *Tj>E(Pc) shRNA* testes, N = 21), Bam expression was detected in excess germ cells (S4B–S4C’ Fig). It has been reported that in *bam* mutant testes, the transition from mitotic spermatogonia to meiotic spermatocyte is abolished, and the testes are enriched with synchronously dividing spermatogonial cells [57,58]. Here the presence of Bam (S4B–S4C’ Fig) and the absence of continuous fusome structure (Fig 2D, S3B and S3B’ Fig) suggest that these excess germ cells in *E(Pc)* somatic knockdown testes had different cellular properties compared to the *bam* mutant.

Even though these excess cells were all positively stained with the germ cell marker Vasa in both *Tj>E(Pc) dsRNA* testes (Fig 2C, 2E and 2E”) and *Tj>E(Pc) shRNA* testes (S3A, S3B’,S3C and S3C” Fig), the early-stage cyst marker Zfh-1 was also detectable in these cells in 12.8% of *Tj>E(Pc) dsRNA* testes (N = 86, Fig 2E’–2E”) and 10% of *Tj>E(Pc) shRNA* testes (N = 79, S3C’–S3C” Fig). To rule out the possibility that these Zfh-1 signals in Vasa-positive cells resulted from background staining of Zfh-1 antibody, *Tj>H2Av-mRFP* control testes (H2Av-mRFP used as a marker) and *Tj>E(Pc) shRNA* testes were co-immunostained and imaged using the same microscopic parameters. Vasa-positive and Zfh-1-positive cells were found in 11% of *Tj>E(Pc) shRNA* testes (N = 45), but not in any of the *Tj-Gal4/H2Av-mRFP* control testes (N = 26). Moreover, germ cells co-stained with Zfh-1 and Vasa were not found in other known germline tumors, such as GSC-like tumor in *nos>upd* testes [8,9] (N = 48, S5A–S5B” Fig) and spermatogonial tumor in *bam*^*1*^*/bam*^*114*^ testes [45,57,58,59] (N = 40, S5C–S5D” Fig). In addition, cells co-stained with Vasa and Yan, another early-stage cyst cell marker, were also found in 13% of *Tj>E(Pc) dsRNA* testes (N = 45, Fig 2F–2F”) and 12% of *Tj>E(Pc) shRNA* testes (N = 43, S3D–S3D” Fig). Our previous studies demonstrate that E(z), a key PcG protein, is required in cyst cells to prevent germ cells from expressing Zfh-1, suggesting a non-cell-autonomous role of E(z) in antagonizing somatic cell fate in the germline [29]. Interestingly, in the present study, compromising E(Pc) function showed phenotypes similar to those resulting from inactivation of E(z) in CySC lineage. Furthermore, when E(z) levels were reduced by either an *E(z)*^*731*^ null allele [29,60] or a deficiency chromosome that uncovers the *E(z)* genomic region at the *Tj>E(Pc) shRNA* background, a more severe excess early-stage germ cell phenotype was observed (S6A–S6D Fig). The similarity of loss-of-function phenotypes and the genetic interactions between *E(Pc)* and *E(z)* are consistent with the previous report that *E(Pc)* acts as an enhancer of PcG mutant [40].

Moreover, although the Tj-Gal4 driver knocks down E(Pc) in all somatic gonadal cells, including hub cells (Fig 1A), knockdown of *E(Pc)* using a hub cell-specific upd-Gal4 driver [61] did not lead to any detectable cellular defect (N = 28, S7 Fig). Notably, these negative data could result from the strength of the upd-Gal4 driver or protein perdurance in the post-mitotic hub cells, which would reduce the efficiency of knockdown effect. Taken together, our data suggest that E(Pc) is required in the CySC lineage to promote germ cell differentiation and antagonize somatic identity in the germline.

In addition to the knockdown strategy, we used the Mosaic Analysis with a Repressible Cell Marker, or MARCM, system [62] to generate *E(Pc)* mutant clones positively labeled by GFP. In control wild-type clones, Zfh-1 was undetectable in 74% (yellow arrows in S8A–S8A”’ Fig) and had diminished signal (yellow arrows in S8B–S8B”’ Fig) in 26% of Eya-positive cyst cells (N = 38). By contrast, Zfh-1 was detectable in all *E(Pc)* mutant cyst cells co-labeled with Eya (N = 49, yellow arrows in S8C–S8C”’ Fig), consistent with overlapping Zfh-1 and Eya expression in the *E(Pc)* knockdown cyst cells shown previously (Fig 1C” and 1F”). Moreover, in 12.5% of testes (N = 64) with *E(Pc)* mutant clones, extra DAPI bright cells were found to intermingle with Zfh-1-positive cells (S8D, S8D’, S8E and S8E’ Fig), resembling the excess early germ cell phenotype observed in *E(Pc)* knockdown testes (Fig 2, S3 Fig). The lower penetrance of the germ cell phenotype using the E(Pc) MARCM clone compared to *E(Pc)* knockdown in the entire CySC lineage likely results from the technical difficulty in ensuring that both cyst cells that encapsulate germ cells are *E(Pc)* mutants. Indeed, in 63% of testes (N = 102) with *E(Pc)* mutant clones, GFP-negative wild-type cyst cells were detectable.

### E(Pc) directly regulates multiple signaling pathway components and the CySC self-renewal factor Zfh-1

**Fig 3:**
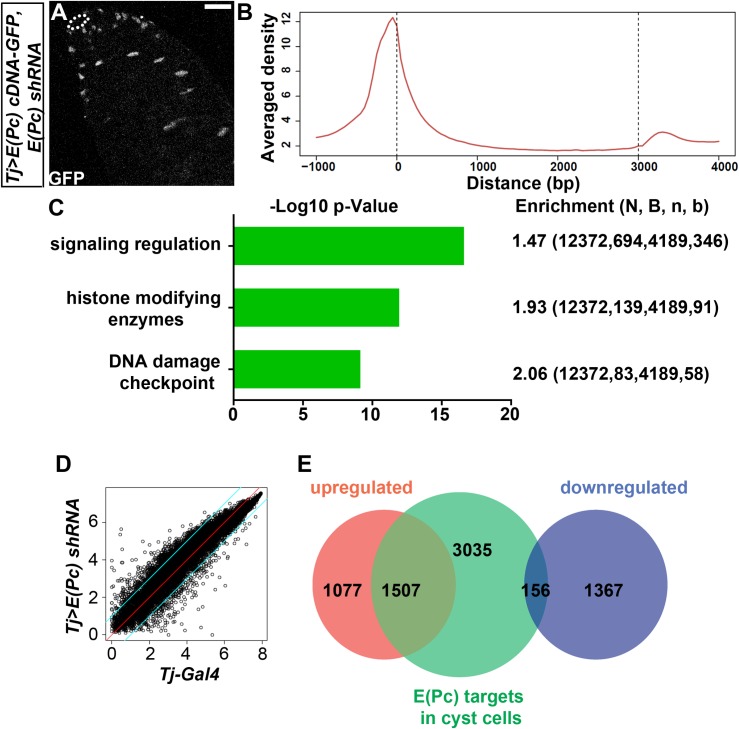
E(Pc) directly regulates multiple signaling pathway components and mainly represses gene expression in cyst cells. (**A**) In *Tj>E(Pc) cDNA-GFP*, *E(Pc) shRNA* testes, GFP is only detectable in CySC lineage. White dotted line: hub. Scale bar: 20μm. (**B**) ChIP-seq was performed with the GFP antibody using *Tj>E(Pc) cDNA-GFP*, *E(Pc) shRNA* testes. Two independent ChIP experiments were performed. Average E(Pc) enrichment signal profile of 4698 genes over a -1-kb to +4-kb region with respect to the transcription start sites (TSSs). (**C**) GO term enrichment test to identify significant categories with distinct biological functions among E(Pc)-binding genes. Enrichment (N, B, n, b): N- total number of genes, B- total number of genes associated with a specific GO term, n- number of all E(Pc) target genes, b- number of E (Pc) target genes with this specific GO term. The scores mean overall enrichment of genes within annotated GO term. -Log 10 P-value annotates the significance of genes enrichment within this specific GO term. (**D**) Scatter plots of gene expression comparison between *Tj-Gal4* control testes and *Tj>E(Pc) shRNA* testes. The two green lines outline differentially expressed genes with more than two-fold change. (**E**) Venn Diagram showing E(Pc) targets upregulated (overlap between red and green) and downregulated (overlap between blue and green) in *Tj>E(Pc) shRNA* testis. See also S9 Fig.

In order to fully understand the molecular mechanisms underlying E(Pc) function in cyst cells responsible for promoting cellular differentiation, a chromatin immunoprecipitation followed by high-throughput sequencing (ChIP-seq) strategy was developed to profile the direct targets of E(Pc) specifically in the CySC lineage. In *Tj>E(Pc) shRNA* testes, a GFP-tagged E(Pc) cDNA transgene was expressed using the same Tj-Gal4 driver. Not only were the *E(Pc)* knockdown phenotypes (Figs 1 and 2, S3 Fig) fully rescued in *Tj>E(Pc) cDNA-GFP*, *E(Pc) shRNA* testes (N = 137), but the E(Pc) cDNA-GFP fusion protein was also exclusively detected in the CySC lineage (Fig 3A). Of note, even though the E(Pc) cDNA-GFP transgene signal was reduced in *Tj>E(Pc) shRNA* testes [S9 Fig, *Tj>E(Pc) cDNA-GFP*, *E(Pc) shRNA* (N = 50) vs. *Tj>E(Pc) cDNA-GFP* (N = 22)], suggesting knockdown effects, we reason that the residual E(Pc) cDNA-GFP is sufficient to rescue the *E(Pc)* knockdown phenotypes. Therefore, this genetic background provided a unique opportunity to immunoprecipitate E(Pc)-bound chromatin in the CySC lineage using a ChIP-grade GFP antibody [63].

We next analyzed our ChIP-seq data to identify the direct targets of E(Pc) in the CySC lineage. When all target genes were plotted over a -1-kb to +4-kb region with respect to the transcription start sites (TSSs), enrichment of E(Pc) could be detected within a 600-bp region upstream of TSSs (Fig 3B), which agrees with the prediction that E(Pc) is a chromatin factor regulating transcription of target genes. Using MACS2 with default setting and the P-value cutoff of 1e-5, we identified 4,698 E(Pc)-bound genes in cyst cells from adult testes. Using the GO term enrichment test [64,65] to further analyze the direct target genes of E(Pc), we found that signaling pathway components, genes responsible for DNA damage checkpoint, and genes encoding histone modifying enzymes represent the top three categories of E(Pc) target genes (Fig 3C). In the signaling pathway category, genes associated with epidermal growth factor (Egf), JAK-STAT, Wnt and Notch signaling pathways are all significantly enriched (P<0.001).

We also performed RNA-seq to compare transcriptomes between *Tj>E(Pc) shRNA* testes and *Tj-Gal4* control testes (Fig 3D). We then interrogated the E(Pc) target genes retrieved from the ChIP-seq dataset with the RNA-seq dataset (S1–S3 Tables). Most of the overlapping genes were upregulated in *Tj>E(Pc) shRNA* testes (1,507 genes) compared with *Tj-Gal4* testes (Fig 3E), suggesting that the normal function of E(Pc) is to suppress transcription. Noticeably, ChIP-seq experiments were performed specifically in cyst cells. At the same time, however, it is extremely challenging from a technical point of view to isolate cyst cells to perform cell type-specific RNA-seq analysis as a result of the tight association between cyst cells and germ cells. Our RNA-seq experiments were therefore performed using the whole testes which reflected transcriptome changes in both germ cells and cyst cells. Notwithstanding, because we could not pinpoint the cyst cell-specific genes that are bound by E(Pc) and have transcriptional change upon knockdown of *E(Pc)*, we focused on a few known genes expressed in cyst cells for further analyses.

**Fig 4:**
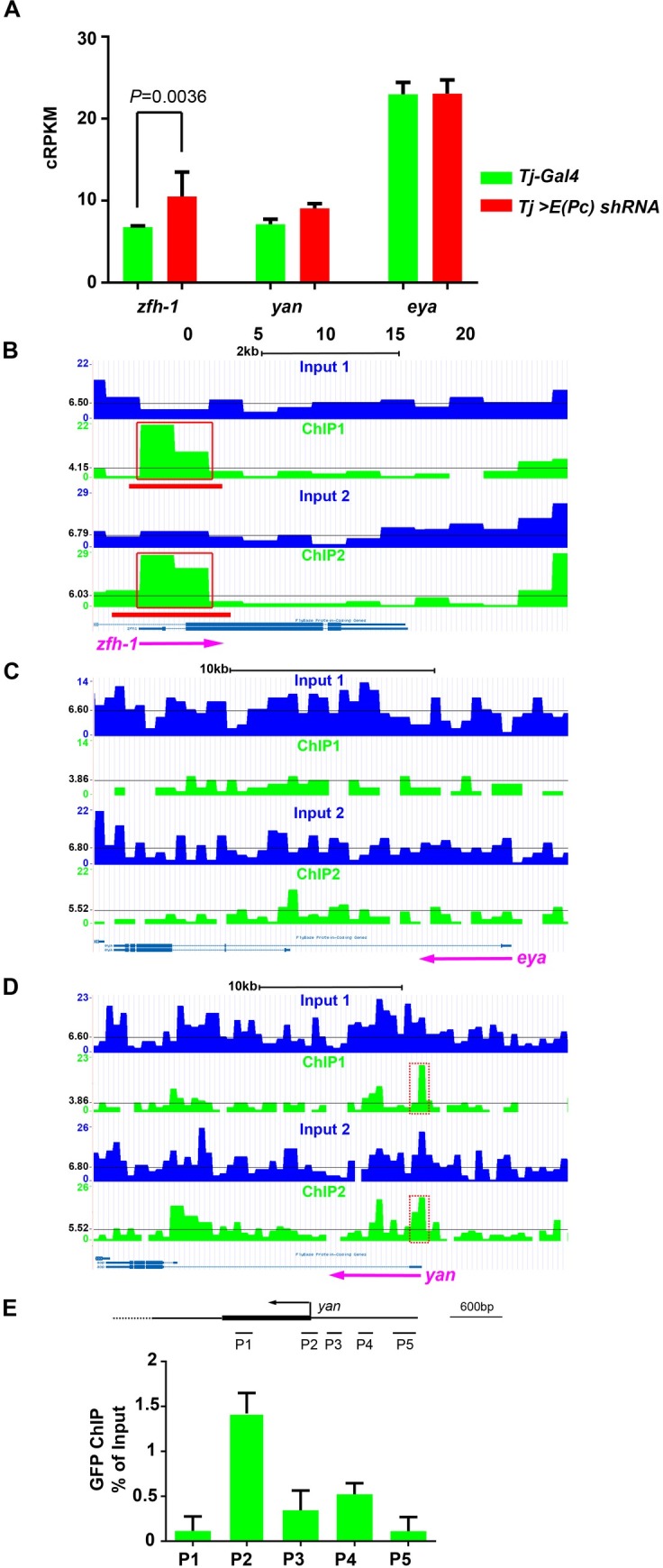
E(Pc) directly regulates the CySC self-renewal factor Zfh-1. (**A**) Expression levels of *zfh-1*, *yan* and *eya* in *Tj-Gal4* control testis and *Tj>E(Pc) shRNA* testis. cRPKM: corrected RPKM (see EXPERIMENTAL PROCEDURES). Error bars, s.d. for N = 2 biological replicates. *P* value: two-tailed t test. (**B**) A genome browser snapshot of the *zfh-1* gene region. Compared with the input control, anti-GFP immunoprecipitated chromatin from cyst cells show enrichment around *zfh-1* promoter region, indicated by red lines below read density map and red box. The black line indicates average read density of chromosome 3R. **(C)** A genome browser snapshot of the *eya* gene region. No enrichment around promoter region is observed. The black line indicates average read density of chromosome 2L. **(D)** A genome browser snapshot of the *yan* (also known as *aop)* gene region. Local enrichment (labeled by red box with dashed line) could be detected around promoter region, although the enrichment did not pass the threshold using peak MACS2 calling algorithm. The black line indicates average read density of chromosome 2L. (**E**) Anti-GFP ChIPed DNA using *Tj>E(Pc) cDNA-GFP*, *E(Pc) shRNA* testes was analyzed by qPCR. Enrichment of E(Pc) at *yan* loci was presented as percentage of input. Error bars, s.d. for N = 2 biological replicates. See also S10 Fig.

The *zfh-1* gene was among the 1,507 upregulated genes (1.64-fold upregulation, P< 0.01, Fig 4A). This is consistent with the excess of Zfh-1-positive cells, as detected by immunostaining in *Tj>E(Pc) RNAi* testes (Fig 1C and 1F”). Enrichment of E(Pc) was found at the endogenous *zfh-1* gene locus (Fig 4B), suggesting that E(Pc) directly binds to and downregulates *zfh-1* expression in cyst cells. By way of contrast, no change in *eya* mRNA level was detected (Fig 4A), in agreement with the immunostaining results showing no change of Eya protein level in *Tj>E(Pc) RNAi* testes (Fig 1C’ and 1F’). Congruent results showed that no E(Pc) enrichment was detected at the endogenous *eya* gene locus (Fig 4C). It may be recalled that Yan, the other early-stage cyst cell marker, showed ectopic expression in *Tj>E(Pc) RNAi* testes (Fig 1D and 1E). Here, E(Pc) binding at the endogenous *yan* locus (Fig 4D) did not pass the cutoff using a peak calling algorithm [66]. To further analyze the potentially weak binding of E(Pc) at the *yan* genomic locus (Fig 4D), ChIPed DNA was analyzed using quantitative PCR (qPCR) with a series of primer sets (Fig 4E) spanning over a 1.8-kb genomic region around the TSS region of the *yan* gene. Compared to the more upstream and downstream sequences, enrichment of E(Pc) could be detected near TSS (Fig 4E). Thus, it is possible that *yan* is a weaker E(Pc) target gene compared to *zfh-1*. This speculation is supported by the slight increase of *yan* detected in *Tj>E(Pc) shRNA* testes (1.28-fold upregulation, P = 0.08, Fig 4A).

If E(Pc) acts as a transcriptional repressor to downregulate *zfh-1* expression, we then reasoned that overexpression of E(Pc) could lead to decreased Zfh-1 levels. Because Zfh-1 is required for CySC self-renewal and GSC maintenance [11], reduction of Zfh-1 might result in loss of both CySCs and GSCs. Indeed, when E(Pc) was overexpressed in the CySC lineage using *Tj>E(Pc) cDNA*, Zfh-1-positive CySCs and early cyst cells were significantly reduced (S10A–S10C Fig). A reduced number of Zfh-1 cells may lead, in turn, to reduction of the cells in the CySC lineage, as shown by decreased Tj-positive cells (S10E–S10G Fig). GSCs also showed a significant decrease (S10A, S10B and S10D Fig), corroborating a previous study reporting that Zfh-1 regulates GSC self-renewal non-cell-autonomously [10]. Collectively, our results showed that E(Pc) is both necessary and sufficient to repress *zfh-1* expression in the CySC lineage.

### E(Pc) acts in synergy with the EGF signaling pathway

Signaling pathway genes comprise the top ontological category of E(Pc) targets (Fig 3C), suggesting their important roles in coordinating crosstalk between somatic and germline lineages. A previous RNAi screen using *Drosophila* S2R+ cells has identified E(Pc) as a positive regulator of the receptor tyrosine kinase and ERK signaling pathway [67]. Our data showed that *yan* is a potential target of E(Pc) (Fig 4D and 4E), which is normally repressed by E(Pc) (Fig 1E and 1G”). Because Yan functions as an antagonist of EGF signaling [46,47], E(Pc) could be a positive regulator of EGF signaling in the CySC lineage. We next studied the potential synergistic activities between E(Pc) and the EGF signaling pathway.

**Fig 5:**
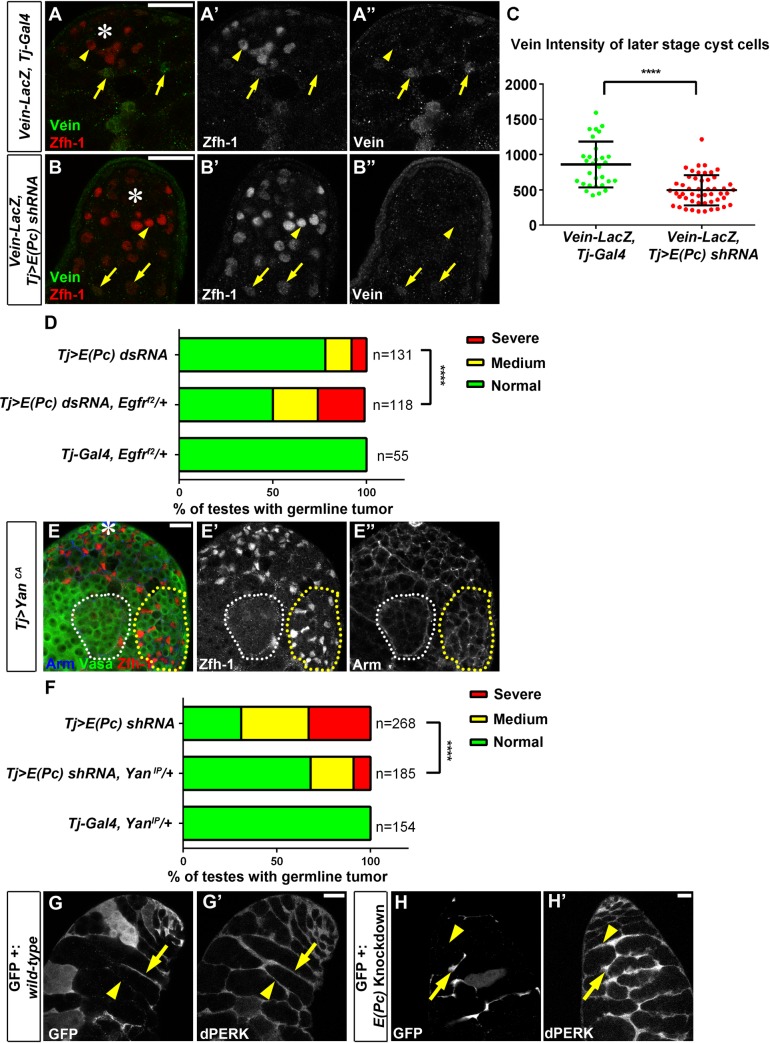
E(Pc) acts in synergy with EGF signaling pathway to promote cellular differentiation in both CySC and GSC lineages. (**A-A”**) In *Vein-LacZ*, *Tj-Gal4* control testes, Vein is not expressed in Zfh-1 positive CySCs (yellow arrowhead), but becomes detectable in differentiated cyst cells (yellow arrows). (**B-B”**) In *Vein-LacZ*, *Tj> E(Pc) shRNA* testes, Vein is not expressed in CySCs (yellow arrowhead), almost undetectable in later stage cells (yellow arrows) several cell diameter away from hub (Asterisk). (**C**) Quantification of Vein expression in later stage cyst cells (labeled by yellow arrows in **A”** and **B”**). ****P<0.0001. Two-tailed t test. (**D**) Quantification of percentage of testes with expanded DAPI bright region (severe, medium and normal, refer to S6A–S6C Fig) in *Tj>E(Pc) dsRNA*, *Tj>E(Pc) dsRNA*, *Egfr*^*f2*^*/+* and *Tj-Gal4*, *Egfr*^*f2*^*/+* testes 3 days after shifting to 29°C. Removing one copy of *Egfr* using loss-of-function *Egfr*^*f2*^ allele enhanced the phenotype. ****P<0.0001, chi-square test. (**E-E”**) Ectopic expression of consistent active (CA) Yan in cyst cells caused overpopulation of Zfh-1 cells (red in **E**, **E’**) accompanying GSC- and GB-like tumor (yellow dashed line) and spermatogonial tumor (white dashed line) within spermatogonial cyst shown by anti-Armadillo immunostaining (**E”**). (**F**) Quantification of percentage of testes with expanded DAPI bright region in *Tj>E(Pc) shRNA*, *Tj>E(Pc) shRNA*, *Yan*^*IP*^*/+* and *Tj-Gal4*, *Yan*^*IP*^*/+* testes 5 days after shifting to 29°C, using the same criterion as in (**D**). Removing one copy of *yan* using a null allele *yan*^*IP*^ suppressed the phenotype. ****P<0.0001, chi-square test. **(G-G’)** Level and localization of phosphorylated ERK (dpERK) in GFP-positive (yellow arrow) and GFP-negative wild-type cells (yellow arrowhead). **(H-H’)** Level and localization of dpERK had no detectable difference between *E(Pc)* knockdown cyst cells (GFP-positive, yellow arrow) and control cyst cells with normal E(Pc) (GFP- negative, yellow arrowhead). Asterisk: hub. Scale bar: 20μm.

The EGF signaling pathway has previously been shown to control the encapsulation of germ cells by cyst cells and then regulate their proper differentiation [16,17,18,19,20,22,68,69]. Consistent with their synergistic activities, knockdown of *E(Pc)* in cyst cells resulted in phenotypes resembling those caused by loss-of-function of EGF signaling pathway components. For example, when EGF signaling is compromised, it has been reported that germ cells have differentiation defects [16,17,18,23,24] and divide asynchronously [22], similar to those germline phenotypes in the *Tj>E(Pc) RNAi* testes (Fig 2D–2D”, S3B and S3B’ Fig). In addition, using the Vein-LacZ reporter as a readout of EGF signaling activity [16,24,70,71,72], expression of this reporter was absent in early-stage Zfh-1-positive cyst cells (yellow arrowhead in Fig 5A–5A”), but expression was robust in differentiated cyst cells (yellow arrows in Fig 5A–5A”), suggesting increased EGF signaling activity during normal cyst cell differentiation, as reported previously [22]. However, in *Tj>E(Pc) shRNA* testes, Vein-LacZ expression was almost undetectable in later stage cyst cells (yellow arrows in Fig 5B–5B”), suggesting compromised EGF signaling activity by *E(Pc)* knockdown. Quantification of the intensity of Vein-LacZ signal in later stage cyst cells (yellow arrows in Fig 5A–5A”, 5B-B”) showed significant difference between *Tj-Gal4* and *Tj>E(Pc) shRNA* testes (Fig 5C, Materials and Methods). Moreover, consistent with the synergistic activities between E(Pc) and the EGF signaling, halving the level of EGFR using a *Egfr*^*f2*^ null allele as heterozygotes enhanced the germline phenotype in *Tj>E(Pc) dsRNA* testes (Fig 5D). On the other hand, a constitutively active form of Yan (Yan^CA^), when expressed in cyst cells using the Tj-Gal4 driver, resulted in phenotypes similar to those observed in *Tj>E(Pc) RNAi* testes (Figs 1C, 2C and 2D, S3A, S3B and S3B’ Fig). First, Zfh-1-positive cells were overpopulated (Fig 5E’) in all *Tj>Yan*^*CA*^ testes (N = 21). Second, excess germ cells were detected as GSC- or GB-like tumors (yellow outline, Fig 5E–5E”) in all *Tj>Yan*^*CA*^ testes (N = 21) and spermatogonial tumors (white outline, Fig 5E–5E”) in 90% of *Tj>Yan*^*CA*^ testes (N = 21). Third, a null allele *yan*^*IP*^ [73] acted as a strong suppressor of the germline differentiation defects in *Tj>E(Pc) shRNA* testes (Fig 5F), supporting the hypothesis that part of the *E(Pc)* knockdown phenotype results from upregulated expression of Yan.

Activated EGF signaling has been shown to induce the entry of phosphorylated active MAP kinase (dpERK) to the nucleus in order to regulate the transcription of target genes [16,68,74]. Therefore, to further understand how E(Pc) regulates EGF signaling, we characterized the expression level and localization of dpERK in *E(Pc)* knockdown cyst cells. We induced *E(Pc)* knockdown and GFP-positive cells using the strategy discussed above (Fig 1F–1G”, Materials and Methods). As a control, GFP-positive wild-type cells were also induced using the same method. In neither case was the level, or subcellular localization, of dpERK distinguishable between GFP^+^ and GFP^-^ cyst cells (Fig 5G and 5G’ and Fig 5H and 5H’), suggesting that E(Pc) may act in parallel with, or downstream of, dpERK to regulate the chromatin state of target genes responsive to the EGF signaling.

### E(Pc) represses JAK-STAT signaling in the CySC lineage

**Fig 6:**
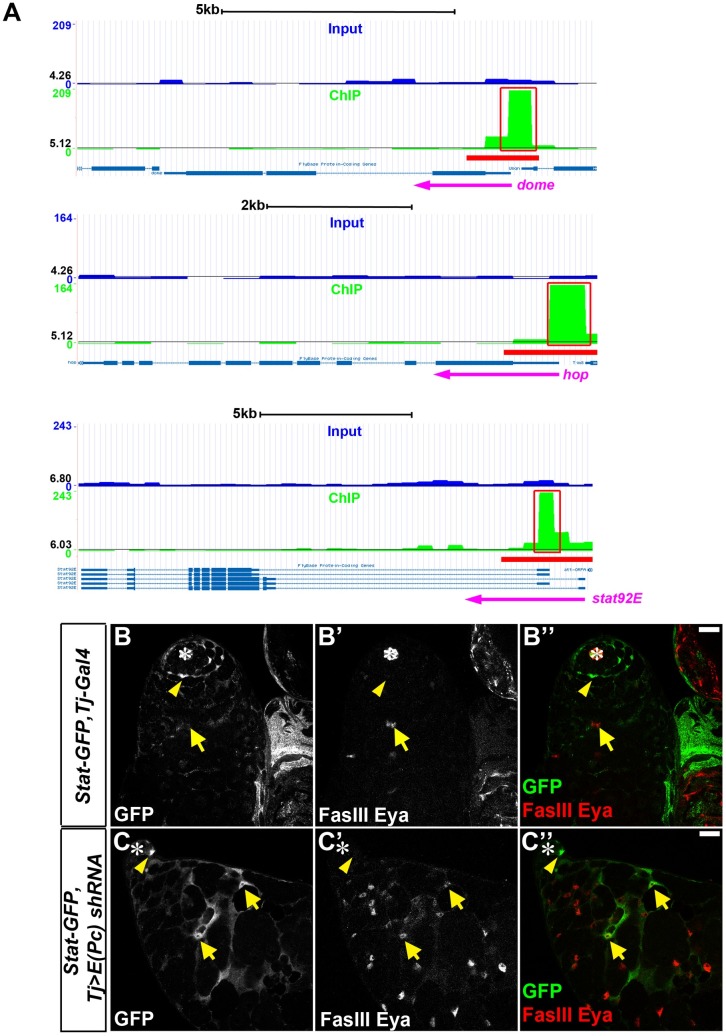
E(Pc) represses JAK-STAT signaling in the CySC lineage. (**A**) Genome browser snapshots of E(Pc) enrichment at *domeless (dome)*, *hopscotch (hop)*, *stat92E* gene loci. One replicate of ChIP experiment is shown here and the other replicate shows similar pattern. Peak calling is labeled by red lines below read density map and also red box. The black line indicates average read density of relative chromosome. (**B-B”**) In *Stat-GFP*, *Tj-Gal4* control testes, GFP signal is enriched in CySCs which are one cell diameter away from hub (asterisk). GFP positive cells (yellow arrowhead) are separable from Eya-positive later stage cyst cells (yellow arrow). (**C-C”**) In *Stat-GFP*, *Tj>E(Pc) shRNA* testes, GFP-positive cells expand from apical CySCs (yellow arrowhead) to Eya-positive cells (yellow arrows). Asterisk: hub. Scale bar: 20μm.

The JAK-STAT signaling pathway has been shown to play prominent roles in regulating self-renewal of both CySCs and GSCs [8,9,10,11,12,15,23,24,75,76,77,78,79]. The Upd ligand is secreted locally from the hub cells and acts through the Domeless receptor to activate the Janus kinase Hopscotch and phosphorylate the STAT92E transcription factor, which is subsequently translocated to the nucleus to activate target gene transcription [80,81]. Our ChIP-seq data identified significant enrichment of E(Pc) at the genomic loci of multiple JAK-STAT pathway genes, including *domeless*, *hopscotch* and *stat92E* (Fig 6A), suggesting that E(Pc) might directly regulate the activity of the JAK-STAT signaling pathway.

We then used a 2X STAT-GFP reporter [28,82,83,84], having two copies of the STAT92E DNA binding sites from a known JAK-STAT target *Socs36E* upstream of the GFP sequences, as a readout of JAK-STAT signaling activity in cyst cells. In the control testes (N = 27), the GFP signal was only detectable in CySCs localized to a diameter of one cell away from the hub region (arrowheads in Fig 6B–6B”), but not in differentiated cyst cells labeled with Eya (arrows in Fig 6B–6B”). By contrast, in 77% of *Tj>E(Pc) shRNA* testes (N = 52), Eya-positive cells showed a robust GFP signal (arrows in Fig 6C–6C”), indicating active JAK-STAT signaling in these later stage cyst cells. Ectopic JAK-STAT activity in the cyst cells with compromised E(Pc) supports the hypothesis that E(Pc) represses JAK-STAT signaling. As shown previously, *zfh-1*, another JAK-STAT signaling target gene [10], also showed ectopic expression in later stage cyst cells in *Tj>E(Pc) RNAi* testes (Fig 1C and 1F”). In summary, these data support the idea that E(Pc) directly represses JAK-STAT signaling activity in the CySC lineage.

### E(Pc) acts in synergy with the Tip60 HAT enzyme in the CySC lineage

*Drosophila* E(Pc) was identified as a component of the Tip60 HAT complex in S2 cells [85]. Biochemical experiments demonstrate that Tip60 acetylates H4 and H2A and that such activity is conserved from yeast [31] to human [86].

**Fig 7:**
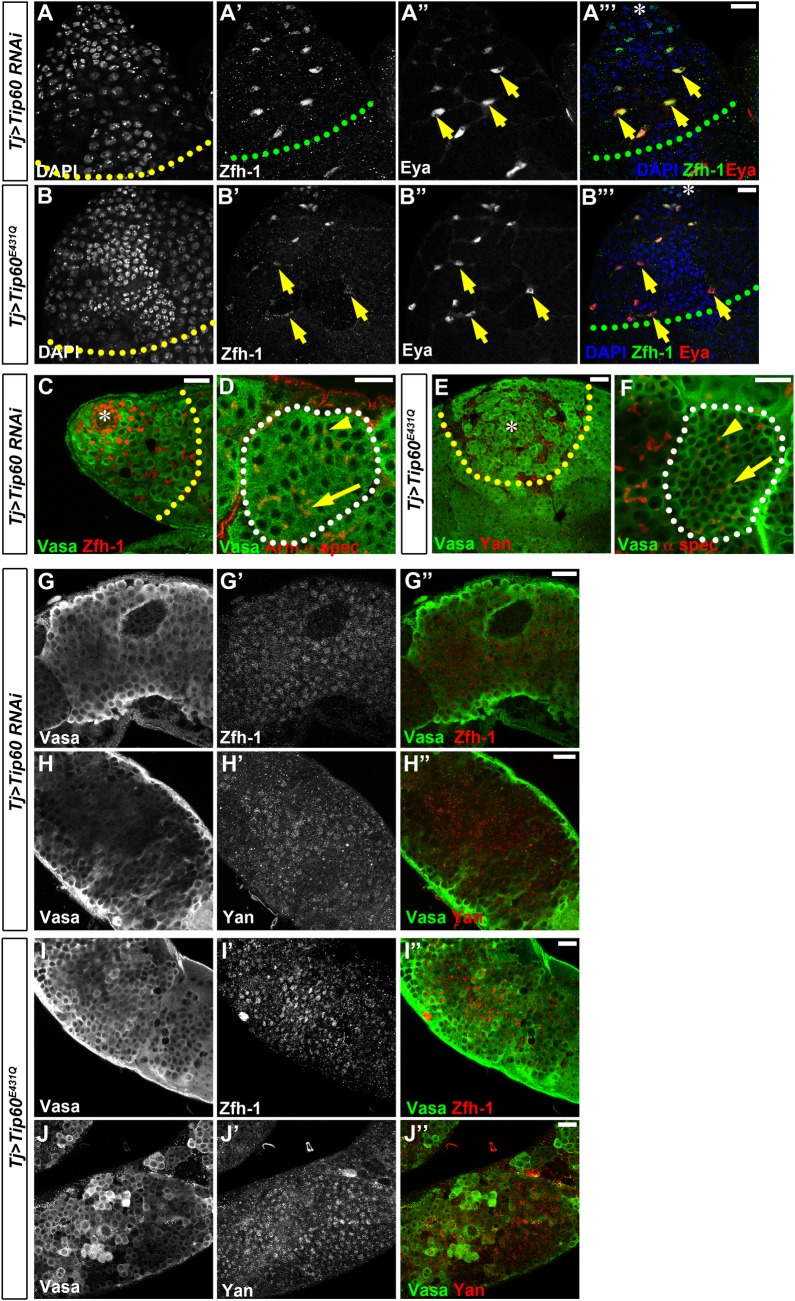
E(Pc) acts in synergy with Tip60 whose function in cyst cells depend on its histone acetyl-transferase activity. (**A-A”‘**) *Tj>Tip60 RNAi* testes show expansion of cells with DAPI bright nuclei (yellow dashed line in **A**) and Zfh-1-positive cyst cells (green dashed line in **A’** and **A”‘**). Zfh-1 is co-expressed with late-stage cyst marker Eya (yellow arrows). (**B-B”‘**) Tip60 HAT deficient dominant active form (*Tip60*^*E431Q*^) expressed using *Tj-Gal4*. *Tj>Tip60*^*E431Q*^ testes show expansion of cells with DAPI bright nuclei (yellow dashed line in **B**), including cells co-expressing Zfh-1 and Eya (yellow arrows in **B’-B”‘**). (**C**) In *Tj>Tip60 RNAi* testes, GSC- and GB-like cells are intermingled with Zfh-1-positive cells (cells from the hub region to yellow dashed line). (**D**) Spermatogonial tumor with more than 16 germ cells (Vasa positive) within one cyst (white dashed line) interconnected by both branched fusome (yellow arrow) and round spectrosome (yellow arrowhead) in *Tj>Tip60 RNAi* testes. (**E-F**) Similar GSC- and GB-like tumor (cells from hub region to yellow dashed line in **E**) and spermatogonial tumor (white dashed line in **F**) are also detected in *Tj>Tip60*^*E431Q*^ testes.(**G-H”**) In *Tj>Tip60 RNAi* testes, Vasa-positive cells (**G**, **G”, H**, **H”**) also have expression of CySC-enriched marker Zfh-1 (**G’**,**G”**) and Yan **(H’**, **H”)**. (**I-J”**) In *Tj>Tip60*^*E431Q*^ testes, Vasa-positive cells (**I**, **I”**, **J**, **J”**) also have expression of Zfh-1 (**I’,I”**) and Yan (**J’**,**J”**). Asterisk: hub. Scale bar: 20μm. See also S11 and S12 Figs.

In order to examine how Tip60 and E(Pc) cooperate in the CySC lineage, we first examined loss-of-function phenotypes of *Tip60* using two strategies: a *Tip60* RNAi transgene [87] (*Tj>Tip60 RNAi*) and a *Tip60* dominant negative form (*Tj>Tip60*^*E431Q*^) [88], both driven by the same Tj-Gal4 as that used in *E(Pc)* knockdown experiments (Figs 1 and 2). We found that both strategies led to defects similar to the phenotypes characterized in *Tj>E(Pc) RNAi* testes. First, excess Zfh-1-expressing cells could be detected in 74% of *Tj>Tip60 RNAi* (N = 70, Fig 7A’) and 77% of *Tj>Tip60*^*E431Q*^ (N = 52, Fig 7B’) testes, leading to the co-expression of Zfh-1 and Eya in the cyst cells of both *Tj>Tip60 RNAi* (yellow arrows, Fig 7A’–7A”’) and *Tj>Tip60*^*E431Q*^ (yellow arrows, Fig 7B’–7B”‘) testes. Second, in 46% of *Tj>Tip60 RNAi* (N = 70, Fig 7A) and 50% of *Tj>Tip60*^*E431Q*^ (N = 52, Fig 7B) testes, expansion of germ cells with DAPI bright nuclei was detected. Further characterization of the excess germ cells showed early-stage germline tumor (Fig 7C) in 8% and spermatogonial tumor (white dotted outline, Fig 7D) in 28% of *Tj>Tip60 RNAi* testes (N = 60), respectively. Similar early-stage germline tumor (Fig 7E) and spermatogonial tumor (white dotted outline, Fig 7F) were also found in 8% and 47% of *Tj>Tip60*^*E431Q*^ testes (N = 38), respectively. Third, cells with both germline marker Vasa and early cyst cell marker Zfh-1 could be detected in 18% of *Tj>Tip60 RNAi* (N = 60, Fig 7G–7G”) and 19% of *Tj>Tip60*^*E431Q*^ (N = 21, Fig 7I and 7I’) testes, respectively. Cells co-expressing Vasa and Yan, another early-stage cyst cell marker, were also observed in 10% of *Tj>Tip60 RNAi* (N = 60, Fig 7H–7H”) and 29% of *Tj>Tip60*^*E431Q*^ (N = 38, Fig 7J–7J”) testes, respectively. Because the mutation in the *Tip60*^*E431Q*^ transgene abolishes the HAT activity of Tip60 [89,90], similar phenotypes between *Tj>Tip60 RNAi* and *Tj>Tip60*^*E431Q*^ testes demonstrate that the function of Tip60 in the CySC lineage relies on its HAT enzymatic activity. In summary, both cyst cell and germline defects in either *Tj>Tip60 RNAi* or *Tj>Tip60*^*E431Q*^ testes were similar to those found in *Tj>E(Pc) RNAi* testes (Fig 1B–1E and Fig 2 and S3 Fig), suggesting that E(Pc) and Tip60 act together to regulate cyst cell differentiation cell-autonomously, as well as coordinate germ cell differentiation and maintain germline fate non-cell-autonomously.

To further explore the potential synergistic activities between E(Pc) and Tip60, we tested their genetic interactions. Because knockdown efficiency using the Gal4: UAS system depends on temperature [91,92,93], flies raised at 25°C instead of 29°C showed less severe phenotypes and with lower penetrance. For example, germline tumor was detected in 0% of *Tj>Tip60 RNAi* (N = 36) and 13% of *Tj>E(Pc) shRNA* (N = 30) testes, respectively (S11A Fig). By contrast, under the same condition, 31% of *Tj>Tip60 RNAi*, *E(Pc) shRNA* testes (N = 39) showed germline tumor phenotype (S11A Fig). In the same way, an *E(Pc)*^*w3*^ mutant [94,95], used as heterozygotes, enhanced the germline phenotype in *Tj>Tip60*^*E431Q*^ testes (S11B Fig).These data suggest that E(Pc) and Tip60 act together in the CySC lineage to regulate germ cell differentiation. It was also notable that overexpression of Tip60 led to significant reduction of Zfh-1-positive cells (S11C Fig) and GSCs (S11D Fig), similar to the effects caused by overexpression of E(Pc) (S10C and S10D Fig). In summary, Tip60 resembles E(Pc) in its necessary and sufficient roles in repressing *zfh-1* expression in the CySC lineage.

Furthermore, if E(Pc) acts with Tip60 whose functions depend on its HAT activity, it is possible that E(Pc) regulates the histone H4 acetylation (H4 ace) state of its target genes. To examine this possibility, anti-H4 ace [96,97] ChIP-ed DNA from both *Tj-Gal4* and *Tj >E(Pc) RNAi* testes were analyzed using qPCR with two series of primers spanning over the genomic loci of *zfh-1* and *yan*, respectively. We found decreased H4 ace at both *zfh-1* (S12A Fig) and *yan* (S12B Fig) genomic regions in *E(Pc)* knockdown testes compared with the control testes, suggesting that the activity of E(Pc) is also required for histone acetylation state at target genes.

## Discussion

**Fig 8:**
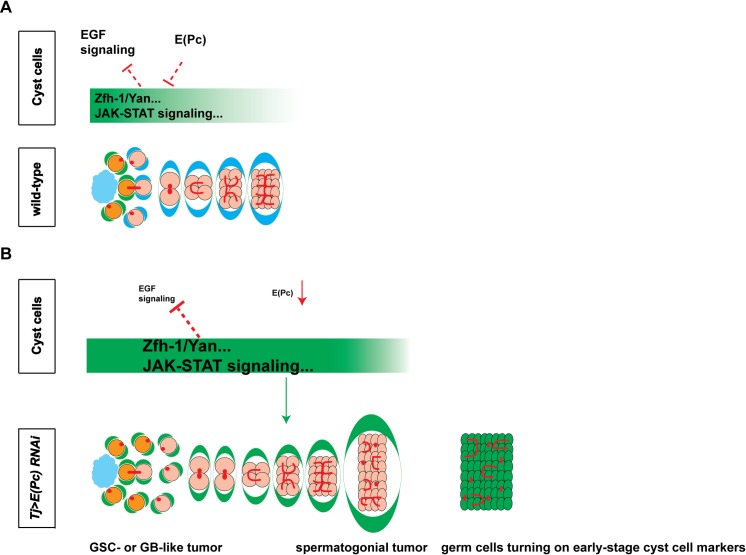
A model to describe both the cell-autonomous functions of E(Pc) in CySC lineage and its non-cell-autonomous roles in regulating germ cell differentiation and maintaining germline identity. (**A**) In cyst cells of wild-type testes, E(Pc) represses CySC-enriched factors (for example Zfh-1, Yan) and JAK-STAT signaling pathway to promote cyst cell differentiation cell-autonomously. E(Pc) also acts in synergy with the EGF signaling pathway. Under this condition, both cyst cell and germ cell differentiate properly. (**B**) When *E(Pc)* is knocked down in cyst cells, ectopic expression of Zfh-1 and Yan, as well as hyperactivation of the JAK-STAT signaling but compromised EGF signaling activity lead to germ cell tumors including both GSC- or GB-like tumor and spermatogonial tumor. In addition, the excess germ cells turn on early-stage cyst cell markers.

In the *Drosophila* testicular niche, the CySC lineage has been thought to play a supportive role for germ cell proliferation and differentiation. However, the mechanisms that explain regulation of CySC lineage differentiation and its coordination with germline lineage have not been elucidated. Here we show that a PcG component, E(Pc), is required for CySC differentiation and that it promotes, in turn, germ cell differentiation. E(Pc) is also required to maintain germ cell identity (Fig 8A). Loss of this critical chromatin regulator in the CySC lineage leads to accumulation of early germ cell tumors, some of which activated the expression of several somatic cell markers tested (Fig 8B). To understand the molecular mechanisms of E(Pc), we performed ChIP-seq experiments to specifically identify E(Pc)-bound targets in somatic gonadal cells. E(Pc) was found to bind many important genes known to be functional in somatic gonadal cells. In particular, E(Pc)-binding genes are enriched with signaling pathway components. Analyses of E(Pc) targets not only confirm some regulatory mechanisms known to coordinate CySC and GSC lineages, such as the EGF signaling, but they also shed light on some new mechanisms. For example, we identified that a direct target of E(Pc) is the *zfh-1* gene. In line with published biochemical results [85], our studies also revealed that E(Pc) works with Tip60 in a HAT-dependent manner in the CySC lineage. The instructive roles of cyst cells in guiding germline differentiation reported here are congruent with the previous finding that genetic ablation of cyst cells leads to germ cell differentiation defects [98].

### E(Pc) acts with the Tip60 HAT enzyme potentially through acetylation of H4

*Drosophila* E(Pc) has been shown to be a component of the Tip60 HAT complex [85]. However, the functional relationship between E(Pc) and Tip60 *in vivo* has not been elucidated. We found that either knockdown of *Tip60* or compromise of HAT activity of Tip60 resulted in phenotypes similar to those caused by E(Pc) loss-of-function (Fig 7). Moreover, enhancement of the *E(Pc)* phenotypes by *Tip60* mutations (S11A and S11B Fig) suggests that E(Pc) acts with Tip60. Finally, the levels of H4 acetylation at *zfh-1* and *yan* genomic regions decrease upon *E(Pc)* knockdown (S12 Fig), suggesting that E(Pc) is required for the HAT activity of Tip60.

Generally, histone acetylation has been linked to gene activation. However, both *zfh-1* and *yan* are upregulated (Fig 4A; Fig 1C” and 1F”; Fig 1E and 1G”) with decreased H4 acetylation levels (S12 Fig) upon inactivation of E(Pc). These data suggest that histone acetylation may repress gene expression. In this regard, it is noteworthy that the antibody against H4 acetylation used for ChIP-qPCR assay is not specific for a particular Lys residue. We speculate that the acetylation of the Lys12 of H4 (H4K12ac) might contribute to this phenomenon. It has been reported that H4K12ac is not associated with the active transcription region in early spermatocytes [99] and is enriched at the chromocentric heterochromatin region at polytene in salivary gland cells [100] in *Drosophila*. Moreover, Tip60 was reported to repress expression of differentiation genes to maintain pluripotency of mouse embryonic stem cells [101], indicating histone acetylation as a contributor to gene silencing. In fact, histone acetylation at other Lys residues was also reported to have repressive roles of gene expression. For example, H3K56ac was reported to repress transcription of newly replicated DNA in budding yeast [102]. Another example is H4K20ac, which was found to be enriched with transcriptional repressors at silenced genes in human cells [103]. Taken together, these data indicate that histone acetylation is not always associated with gene activation, but that it could contribute to gene silencing.

### Germline defects by inactivating a chromatin regulator in the neighboring somatic cells

Even though knockdown of E(Pc) in CySC lineage leads to CySC differentiation defects, the most prominent phenotypes were detected in the germline. Germ cells in *Tj>E(Pc) RNAi* testes have interesting new phenotypes. First, excess germ cells divide asynchronously, a phenotype different from previously identified spermatogonial tumors in *bam* and *benign gonial cell neoplasm* (*bgcn*) mutant testes, in which the transition from spermatogonia to spermatocyte is abolished, and the testes are enriched with synchronously dividing spermatogonia [57,58]. However, the presence of Bam (S4B–S4C’ Fig) and the absence of continuous fusome structure (Fig 2D, S3B and S3B’ Fig) both suggest that these excess germ cells in *Tj>E(Pc) RNAi* testes have different cellular properties. In addition, the excess germ cells do not resemble expanded early-stage germ cells upon hyperactivation of the JAK-STAT signaling pathway in *Drosophila* testis [8,9,10]. In fact, the excess germ cells in *Tj>E(Pc) RNAi* testes have features resembling both spermatogonial tumors and GSC- or GB-like tumors (Fig 2 and S3 and S4 Figs).

Second, germ cells in *Tj>E(Pc) RNAi* testes ectopically turn on early-stage cyst cell markers, such as Zfh-1 and Yan, indicating that E(Pc) acts in cyst cells to prevent germ cells from taking somatic cell fate. The dichotomy between germline and soma represents the earliest lineage specification among many metazoan organisms. In multiple model organisms, including *C*. *elegans* and *Drosophila*, germ cell identity is determined by maternally loaded germ granules [104,105,106,107,108,109]. After specification, protection of germline fate requires both proper chromatin state and specific cytoplasmic factors in the germ cells [110,111,112,113,114,115,116,117]. Previous study identified PcG component E(z) as a non-cell autonomous factor in repressing the somatic fate of germ cells in adult *Drosophila* testis [29]. Here we identified that both E(Pc) and Tip60 play similar roles in cyst cells to maintain the germline identity in a non-cell autonomous manner, indicating that they might act with E(z) in regulating a critical signaling pathway (or pathways) to keep germline identity throughout adulthood. These results also emphasize the important roles of the somatic gonadal cells in protecting germline from taking somatic cell fate to ensure proper differentiation into functional gametes.

### E(Pc) is a potential master regulator of multiple signaling pathways for the communications between CySC and GSC lineages

Even though previous studies suggest that somatic gonadal cells control germ cell differentiation and maintain germline identity through multiple signaling pathways [8,9,11,16,17,29,49,76,98,118,119,120], it is unclear how these signaling pathways themselves are regulated. Here our ChIP-seq results reveal that E(Pc) is enriched at key components of multiple signaling pathways known to be important in regulating germ cell function. For example, the EGF signaling pathway has been shown to regulate cyst cells in encapsulating germ cells and promoting their proper differentiation in *Drosophila* testis [16,17,18,19,20,22,68,69]. We found that the EGF antagonist Yan is highly enriched in CySCs, but decreased dramatically in later stage cyst cells repressed by E(Pc) during CySC differentiation. In line with this, the expression of Vein, which is downstream of the EGF signaling, is compromised in *Tj>E(Pc) RNAi* testes (Fig 5B–5B” and 5C). Recently, decreased EGF signaling has been shown to induce extra germ cell division out of synchrony [22]. Similar asynchronous division of germ cells upon compromising either E(Pc) function or EGF signaling suggests that E(Pc) acts in synergy with the EGF signaling pathway, probably through regulation of the chromatin state at the endogenous *yan* locus.

Similarly, E(Pc) was found to be enriched at multiple JAK-STAT pathway components. However, different from increased EGF signaling activity during CySC differentiation, high JAK-STAT signaling activity is only detectable in early-stage cells of both CySC and GSC lineages [12,14,77,84,121,122]. Hyperactivation of JAK-STAT signaling in either CySC or GSC lineage is sufficient to block cellular differentiation and results in tumors with CySC- and GSC-like features [8,9,10]. In this scenario, E(Pc) might downregulate JAK-STAT signaling to promote CySC differentiation by directly repressing the expression of key JAK-STAT components. Consistent with this finding, we observed that the 2X STAT-GFP reporter showed prolonged expression in later stage cyst cells when E(Pc) was inactivated (Fig 6C–6C”). However, this reporter uses the upstream regulatory sequences from the *Socs36E* gene [82], which itself acts as a repressor of JAK-STAT signaling [123,124]. This negative feedback regulation of JAK-STAT signaling might explain why removal of one copy of stat gene, using null allele *stat*^*06346*^, or *zfh-1* gene, using either a mutant allele *zfh-1*^*75*.*26*^ or a deficiency chromosome that uncovers the *zfh-1* gene region [10,125]), did not efficiently suppress the *Tj>E(Pc) RNAi* phenotype: the percentages of testes with medium and severe excess germ cells were 35% and 37% for *Tj>E(Pc) shRNA* testes (N = 156), 47% and 53% for *Tj>E(Pc) shRNA*, *Stat92E*^*06346*^*/+* testes (N = 75), 34% and 43% for *Tj>E(Pc) shRNA*, *Zfh-1*^*75*.*26*^*/+* testes (N = 155), and 47% and 29% for *Tj>E(Pc) shRNA*, *Df[Zfh-1]/+* testes (N = 150), respectively. Furthermore, studies in the *Drosophila* optic lobe identified E(Pc) as one JAK-STAT target positively regulated by JAK-STAT signaling [126], suggesting mutual regulation between *E(Pc)* and the JAK-STAT signaling.

Collectively, then, we found that E(Pc) regulates multiple signaling pathways and may act as a master regulator for the communications between the somatic and germline lineages in the *Drosophila* adult testis. The ultimate readout in *E(Pc)* mutants depends on the particular E(Pc) targets in the signaling pathway(s) and is complicated by the dual roles of E(Pc) in either activating or repressing gene expression. However, this complicated feature of E(Pc) regulation might be necessary to fine tune activities of different signaling pathways.

In summary, we demonstrate that a chromatin factor E(Pc) acts in cyst cells and is responsible for germline differentiation and germ cell fate maintenance. These results emphasize the importance of the microenvironment where germ cells reside in antagonizing somatic identity and promoting germ cell differentiation. Similar to *Drosophila* testis, many mammalian stem cell niches support multiple stem cells. For example, both hair follicle stem cells and melanocyte stem cells co-occupy the hair follicle bulge [127,128]. The hair follicle stem cells have been shown to function as a niche for melanocyte stem cells through the TGF-β signaling [129]. Similarly, mesenchymal stem cells and hematopoietic stem cells co-exist in the bone marrow, and mesenchymal stem cells constitute the hematopoietic stem cell niche [130]. Understanding the coordination between two stem cell lineages during differentiation may shed light on other complex niches that support multiple stem cell populations.

## Materials and methods

### Fly strains and husbandry

Flies were raised under standard yeast/molasses medium at 25°C unless stated otherwise. The following flies were used: *E(Pc)*^*1*^ (Bloomington Drosophila Stock Center, BL3056), *E(Pc)*^*w3*^ (BL9396), *UAS-E(Pc) dsRNA* (BL28686), *UAS-E(Pc) shRNA* (BL35271), *upd-Gal4* (from D. Harrison, University of Kentucky, Lexington, KY, USA), *Tj-Gal4* (Kyoto stock center, DGRC#104055), *Egfr*^*f2*^(BL2768), *yan*^*IP*^(BL3101), *E(z)*^*731*^ (BL24470), *Df [E(z]* (BL29023), *UAS-yan.ACT* (BL5789), *Vein-lacZ* (BL11749), 2X STAT-GFP [82], *Stat92E*^*06346*^ (from N. Perrimon, Harvard Medical School, Boston, MA, USA), *UAS-Tip60 dsRNA* (BL28563), *UAS-dTip60*^*E431Q*^, *UAS-Tip60* (from Felice Elefant, Drexel University, Philadelphia, Pennsylvania, USA), *Bam-HA* [131], *hs-FLP; Act5c*.*FRT-CD2-FRT*.*Gal4; UAS-GFP* flies (from Allan Spradling, Carnegie Institution for Science, Department of Embryology, Baltimore, Maryland, USA) [132], *UAS-GFP hs-FLP; FRT42D*, *Tub-Gal80; Tub-Gal4* (from Duojia Pan, Johns Hopkins Medical Institution, Baltimore, Maryland, USA), *P{neoFRT}42D* (BL1802), *zfh-1*^*75*.*26*^ (from Ruth Lehmann, NYU school of medicine, New York, USA), *Df(zfh-1)* (BL7917), *UAS-H3GFP*, *UAS-Upd* (from Stephen DiNardo, Perelman School of Medicine at the University of Pennsylvania, Department of Cell and Developmental Biology, Philadelphia, PA, USA), *bam*^*114*^*/TM6B* (from Margaret T. Fuller, Developmental Biology and Genetics, Stanford University School of Medicine, Stanford, CA), *bam1/TM3* (from Allan Spradling, Carnegie Institution for Science, Department of Embryology, Baltimore, Maryland, USA).

To study function of E(Pc) in cyst cells, two independent RNAi lines *UAS*-*E(Pc) dsRNA* and *UAS*-*E(Pc) shRNA* were crossed with different drivers *upd-Gal4* and *Tj-Gal4* at 25°C, respectively. Newly enclosed progenies were shifted to 29°C and maintained for 8–10 days (D) before dissection. For Tip60 function study, RNAi line *UAS*-*Tip60 dsRNA* and dominant negative HAT deficient line *UAS-Tip60*^*E431Q*^ were crossed with *Tj-Gal4* at 25°C and then adult progenies were shifted to 29°C and maintained for 8–10 D before dissection.

To identify if *E(Pc)* genetic interacts with *Egfr*, *yan*, *E(z)*, *Stat92E*, alleles *Egfr*^*f2*^, *yan*^*IP*^, *E(z)*^*731*^, *Stat92E*^*06346*^, *zfh-1*^*75*.*26*^ and deficiency lines *Df [E(z)]*, *Df(zfh-1)* were used. Flies with the following genotypes: *Tj-Gal4/ Egfr*^*f2*^*; UAS-E(Pc) dsRNA/+*, *Tj-Gal4/+; UAS-E(Pc) dsRNA/+* were shifted to 29°C for 3D before analysis. Flies with the following genotypes: *Tj-Gal4/yan*^*IP*^*; UAS-E(Pc) shRNA/+*, *Tj-Gal4/+; E(z)*^*731*^ */ UAS-E(Pc) shRNA*, *Tj-Gal4/+; Df [E(z)]/UAS-E(Pc) shRNA*, *Tj-Gal4/+; UAS-E(Pc) shRNA/ Stat92E*^*06346*^, *Tj-Gal4/+; UAS-E(Pc) shRNA/* *zfh-1*^*75*.*26*^, *Tj-Gal4/+; UAS-E(Pc) shRNA/ Df(zfh-1)* were shifted to 29°C for 5D before dissection.

To study if expression of *E(Pc) cDNA-GFP* in cyst cells is sufficient to rescue *Tj>E(Pc) RNAi* phenotype, flies with the following genotype: *Tj-Gal4/ UAS-E(Pc) cDNA-GFP; UAS-E(Pc) shRNA/+*, *Tj-Gal4/ UAS-E(Pc) cDNA-GFP; UAS-E(Pc) dsRNA/+* were dissected at 5D after shifting from 25°C to 29°C. To test potential defects by overexpression of E(Pc) or Tip60, testes from *Tj-Gal4/ UAS-E(Pc) cDNA*, *Tj-Gal4/ +; UAS-Tip60 cDNA/+* males 10-11D after shifting from 25°C to 29°C were analyzed.

### Generation *E(Pc)* knockdown cyst cell clones and MARCM clones

To analyze function of E(Pc) in individual cyst cells, flies with the following genotype: *hs-FLP; Act5c*.*FRT-CD2-FRT*.*Gal4/+; UAS-GFP/UAS-E(Pc) shRNA* and *hs-FLP; Act5c*.*FRT-CD2-FRT*.*Gal4/+;UAS-GFP/+* (*hs*: *heatshock* promoter, *Actin*: *actin* promoter) were heat shocked at pupal stages for two days with two hours on each day. Enclosed flies were aged for 5-6D after heat shock and used for dissection and immunostaining.

To generate MARCM clones, *E(Pc)*^*1*^ null allele was recombined with *FRT42D* to generate *FRT42D*, *E(Pc)*^*1*^*/Cyo* flies. Adult flies with following genotype: *UAS-GFP hs-FLP; FRT42D*, *Tub-Gal80/ FRT42D*, *E(Pc)*^*1*^*; Tub-Gal4* and control flies *UAS-GFP hs-FLP; FRT42D*, *Tub-Gal80/ FRT42D; Tub-Gal4* were aged for one day, then heat shocked for 2 hours and aged until dissection.

### Transgenic fly lines generation

For transgenic fly *UASp-E(Pc) cDNA* and *UASp-E(Pc) cDNA-GFP*, E(Pc) cDNA was amplified using cDNA prepared from wild-type testis as the template. The 5’ half of E(Pc) cDNA was amplified as a KpnI and NotI flanked fragment with E(Pc) F1 and R1 primers. The 3’ half of E(Pc) cDNA was amplified as an NotI and XbaI flanked fragment using E(Pc) F2 and R2 primers. These two fragments were then ligated into pGEM-T-easy vector (Promega) followed by sequencing. To insert the *GFP* sequences at the 3’-end of *E(Pc)* cDNA, a Pml I site was generated right upstream of the stop codon of *E(Pc)* within R2 primer. *GFP* fragment was amplified as a Pml I and Xba I flanked fragment with Primer 5’ GFP and 3’ GFP, followed by ligation into pGEM-T-E(Pc) 3’ half cDNA opened with Pml I and XbaI restriction enzyme digestion. Finally, the 5’ half *E(Pc)* cDNA in a KpnI to NotI fragment and the 3’ half with and without GFP in a Not I to Xba I fragment were ligated into pBlueScript vector (Agilent Technologies) cut with Kpn I and Xba I in a 3-way ligation to generate a KpnI and XbaI flanked full-length E(Pc) cDNA tagged with GFP. Then the E(Pc) full cDNA tagged with GFP was cut with Kpn I and Xba I and ligated into UASp vector cut using same two enzymes.

To generate E(Pc) genomic plasmid tagged with GFP, a 21 kb P[acman] BAC clone (CH322-140G22) covering the entire *E(Pc)* genomic region was ordered from BACPAC Resources Center (BPRC). Zra I is one unique enzyme site close to the stop codon of *E(Pc)* genomic region. Pac I is another unique enzyme site within the 3’UTR region of *E(Pc)*. Using primers 3’UTR F and 3’UTR R ended with Asc I and Pac I, an approximate 3 Kb fragment was amplified using the BAC clone as template and ligated into pGEM-T easy vector. Using primers GFP F and GFP R ended with Zra I and Asc I GFP sequence was amplified. Then, the GFP in a Zra I to Asc I fragment was ligated into pGEM-T 3’UTR vector, cut with AscI and PacI to generate a GFP-3’ UTR fragment flanked by ZraI and PacI. Then GFP-3’ UTR cut with ZraI and PacI was ligated into P[acman], opened with ZraI and PacI to generate E(Pc) genomic plasmid tagged with GFP.

Transgenic fly lines were generated by Bestgene Inc (Chino Hills, CA). More than three independent transgenic lines were generated for each transgene.

Primers:

E(Pc) F1: GGGGTACCATGTCCAAGCTGTCGTTCAGAGCCC
E(Pc) R1: ATAAGAATGCGGCCGCTGCCGCCGA
E(Pc) F2: ATAAGAGCGGCCGCCGCTCGTGC
E(Pc) R2: GCTCTAGATCACACGTGTCTGTTGATGGTTGACGTCACAC
5’ GFP: ATCACGTGATGGTGAGCAAGGGCGAGGAG
3’ GFP: GCTCTAGATTACTTGTACAGCTCGTCCATGCCG
3’UTR F: GGCGCGCCGACGCGGATGGCAGCG
3’UTR R: CCTTAATTAAACATACATACGTATTCTTTTTGTTTTGG
GFP F: GACGTCAACCATCAACAGAATGGTGAGCAAGGGCGAG
GFP R: GGCGCGCCTTACTTGTACAGCTCGTCCATGC

### Immunostaining

Testes were dissected in 1X PBS and then fixed in 4% formaldehyde in 1X PBS for 30 min at room temperature (RT). Then testes were washed twice with 20min each time using 1X PBST (0.1% triton) at RT. Testes were incubated with Primary antibodies on nutator at 4°C overnight. After twice wash with 1X PBST, testes were incubated with secondary antibodies in darkness at RT for 2 hours. After twice wash with 1X PBST, testes were mounted using Vectashield (Vector H-1200). Primary antibodies used are: Vasa (Rabbit, Santa Cruz, sc-30210), Vasa (Rat, 1:100, developed by Spradling, A. C./ Williams, D. obtained from DSHB), Zfh-1 (Rabbit, 1:5000, from R. Lehmann), Fas III (Mouse, 1:100, DSHB, 7G10), Armadillo (Mouse, 1:200, DSHB, N2 7A1), Eya (Mouse, 1:25, DSHB, 10H6), Tj (Guinea pig, 1:1000, from M. Van Doren), Yan (Mouse, 1:25 after pre-absorption against *Drosophila* embryos, DSHB, 8B12H9), GFP (Chicken, 1:1000, Abcam, ab13970), dpERK (Rabbit, 1:100, Cell signaling, #4370), HA (Rat, 1:50, Roche, 3F10), β-Galactosidase (Mouse, 1:200, Sigma, G4644). For dpERK staining, testes were dissected in 10 mM Tris-Cl pH 6.8, 180 mM KCl, 50 mM NaF, 1 mM Na3VO4, 10 mM b-glycerophosphate as described before [16]. Secondary antibodies were all Alexa Fluor series (1:200, Molecular Probes). Images were taken with Zeiss LSM 510 META or LSM 700. Images were processed using Adobe Photoshop. EdU incorporation was performed with Click-iT EdU Alexa Fluor 488 imaging kit (Invitrogen C10083). Dissected testes were incubated with EdU solution for 30min, followed by fixation and immunostaining as described.

### Quantification of vein intensity and data analysis

To compare Vein intensity between the *Tj-Gal4* control and *Tj>E(Pc) shRNA* testes, H2Av-mRFP (BL34498) transgene was used as a marker to distinguish the two genotyped fly testes. Testes dissected from *Tj-Gal4/H2Av-mRFP; Vein-LacZ/+* males were compared with *Tj-Gal4/+; Vein-LacZ/UAS-E(Pc) shRNA* testes, which were immunostained together and imaged using the same parameters. Control testes could be identified based on the H2Av-mRFP marker. Vein-LacZ fluorescence intensity was measured for each Z stack across the entire nucleus using Image J software and summed up. Data were analyzed and presented using GraphPad Prism software.

### Chromatin immunoprecipitation sequencing (ChIP-seq) and data analysis

Flies with following genotype: *Tj-Gal4/ UAS-E(Pc) cDNA-GFP; UAS-E(Pc)shRNA/+* were collected as newly eclosed males and aged for 5D at 29°C after shift from 25°C. Approximately 2,000 pairs of testis were dissected and grouped into two batches which were used as two replicates for ChIP experiments, which were performed using ChIP-IT high sensitivity kit (#53040, Active motif) following the manufacturer’s instruction. A ChIP-grade GFP antibody (Abcam, ab290) was applied. Sonication of fixed testes was performed using Bioruptor sonicator (UCD-200, diagenode) using the following setting: 0.5min ON, 1min OFF repetitively for a total of 25min. The size of DNA associated with sonicated chromatin was checked which was approximate 400–500 bp. Similar protocol was used for ChIP with H4 tetra acetylation antibody (A gift from Keji Zhao, NHLBI, NIH) and 100 pairs of flies with following genotype: *Tj-Gal4/+;E(Pc) shRNA/+* or *Tj-Gal4/+*.

Libraries were generated using reagents provided in the Illumina TruSeq ChIP Sample Preparation Kit (IP-202-1012). The Illumina compatible libraries were sequenced with Mi-seq desktop sequencer (Mi-Seq, Illumina). Then 75 bp single-end read sequencing was performed. FASTQ raw data files were filtered with quality control software Fastqc (www.bioinformatics.babraham.ac.uk/projects/fastqc/). BOWTIE program [version 0.12.7, [133]] was utilized to align reads to *Drosophila* genome (dm3), with the running parameters (bowtie -p 8 -t -a—phred33 -quals -n 2 -e 70 -l 48 -m 1—best—strata). Single-end reads were treated as separate single reads. At each chromosome position, only one read was retained to get a non-redundant read count data. SAM formatted alignment files will be uploaded onto NIH GEO database upon paper acceptance. Enrichment of reads across the genome was analyzed by MACS2 [66] for peak calling. The peak calling was performed with paired experiment (GFP ChIP) and control genome input under default parameter settings. UCSC genome browser customized visualization tools were also applied in the analysis [134]. SAMtools [135] software suite was utilized to convert between related read formats. Go term analysis was performed using Gorilla [64,65].

### ChIP quantitative PCR (qPCR)

The qPCR experiments were performed as previously described [136]. Two independent biological replicates were used. Each PCR reaction was performed in duplicates and averaged Ct values were used. Primers used for qPCR are listed in S4 Table.

### RNA-seq and data analysis

One pairs of *Tj-Gal4* or *Tj-Gal4/+; UAS-E(Pc) shRNA/+* testes were dissected in PBS, respectively as one replicate. Two replicates were generated for each genotype. Total RNA was purified following the manufacturer’s instruction of PicoPure RNA isolation kit (KIT0204, Life technologies). Then both libraries were generated using reagents provided in Illumina TruSeq RNA Sample Preparation Kit (RS-122-2001). The Illumina compatible libraries were sequenced with Illumina Hiseq2500 sequencer in the high-throughput sequencing core facility at Johns Hopkins University Bayview with 50 bp single-end reads.

For the alignment to fly genome and gene mapping, sequencing reads were examined by fastqc quality control software (http://www.bioinformatics.babraham.ac.uk/projects/fastqc/). The reads which passed quality filter were mapped back to *Drosophila* genome (dm3) (Flybase dmel_r5.43, as of Jan 2012, ftp://ftp.flybase.net/releases/FB2012_01/dmel_r5.43/). Bowtie aligner (version 0.12.7) [133] was utilized with the following configuration (-a—phred33-quals -n 2 -e 70 -l 28 -m 1—best–strata) which allows two mismatches and only one alignment site. We then assigned each read into gene regions. The annotation for protein coding genes were retrieved from Flybase database (as of Jan 2012, ftp://ftp.flybase.net/releases/FB2012_01/dmel_r5.43/). The exons from different alternative splicing isoforms were merged to find the maximum genome coverage regions per gene. When a read is mapped to a region with more than one gene, i.e., one merged exon region overlaps with a non-coding gene, the count is split as equal possibilities into these two genes with half count for each. A matrix file with the number of reads assigned into each gene per sample was prepared for the following data analysis.

To identify differentially expressed genes, we utilized the edgeR software package [137] in *R* to find the normalization factors for each sample with various sizes [by the TMM (Trimmed Mean of M value) and upper quantile normalization methods]. The edgeR method models short reads into negative binomial distribution and estimates the biological replicate variance (dispersion). Tag-wise dispersion estimation was performed in “*Tj-Gal4”*, *“Tj-Gal4/+; E(Pc) shRNA/+”* two groupings of read count profiles. We introduced quantity term “corrected RPKM (cRPKM)” by the formula: pseudo.alt * 1e+09 / (length of merged transcripts)/ (common.lib.size). The common.lib.size was calculated from the calcNormFactors function of edgeR, which performs TMM and upper quantile normalization methods and set a reference library. The pseudo.alt contains read counts after normalization across the input multiple profiles. The pseudo.alt was calculated by edgeR using quantile normalization and maximum likelihood method. The pseudo.alt contains pseudo read counts after correcting the library size and composition differences.

After cRPKM calculation, gene expression levels per sample were pair-wisely compared with spearman correlation (correlation coefficient rho). A pair-wise inter-profile distance was defined as (1-rho) and set up a distance matrix. A dimension reduction method, multidimensional scaling in *R* (http://stat.ethz.ch/R-manual/R-devel/library/stats/html/cmdscale.html), was utilized to visualize the global similarity relationship of 4 samples.

### Accession numbers

GEO accession number for ChIP-seq and RNA-seq data is GSE93828.

## Supporting information

S1 Fig Expression pattern of GFP from a genomic rescuing *E(Pc) gDNA-GFP* transgene in *Drosophila* adult testes. (**A-A”**) GFP signal in the nuclei of both germ cells (yellow arrowheads) and Eya-positive late-stage cyst cells (yellow arrows). Scale bar: 50μm. (**B-B”**) At the apical tip, GFP signal in the nuclei of germ cells (white arrowhead in **B’** and yellow arrowhead in **B”**), early cyst cells (Zfh-1-positive cell, labeled by white arrow in **B’**) and later cyst cells (Eya-positive cell, labeled by yellow arrow in **B”**). (**C-C”**) In *Tj>E(Pc) RNAi*, *E(Pc) gDNA-GFP* testes, undetectable GFP signal in early cyst cells (Zfh-1-positive cell, labeled by white arrow in **C’**) and later cyst cells (Eya-positive cell, labeled by yellow arrow in **C”**), but detectable in germ cells (white arrowhead in **C’** and yellow arrowhead in **C”**). Asterisk: hub. Scale bar: 20μm.
(TIF)

S2 Fig Knockdown of *E(Pc)* in cyst cells led to increased Tj-positive and Eya-positive cells but had no effect on the cell-type or stage-specificity of the *Tj-Gal4* driver. (**A**) Percentage of testes with <10, 10–30 and >30 Zfh-1- and Eya-double positive cyst cells in different genotyped testes. (**B-C**) Immunostaining with anti-Tj and Eya in *Tj-Gal4* and *Tj>E(Pc) shRNA* testes. (**D**) Quantification of Tj-positive cells in *Tj-Gal4* control testes: 50 ± 12.49 (Mean ± SD, N = 40) and in *Tj>E(Pc) shRNA* testes: 83.91 ± 22.41 (N = 31). Quantification of Eya-positive cells at the tip of *Tj-Gal4* control testes: 39 ± 7.35 (Mean ± SD, N = 24) and *Tj>E(Pc) shRNA* testes: 58 ± 13.04 (N = 43). **** *P*<0.0001, two-tailed *t* test. (**E-F’”**) Immunostaining using the germ cell marker Vasa (**E’**, **F’**) and a late cyst cell marker Eya (**E”**, **F”**) in *Tj>H3 GFP* and *Tj> H3 GFP*, *E(Pc) shRNA* testes. Asterisk: hub. Scale bar: 20μm.
(TIF)

S3 Fig Knockdown of *E(Pc)* in cyst cells using a different short hairpin (sh) RNA also led to germ cell overproliferation and ectopic expression of cyst cell markers. Immunostaining using the germ cell marker Vasa (**C** and **D**, green in **A**, **B’**, **D”**), early cyst cell markers Zfh-1 (**C’**, red in **A**, **C”**) and Yan (**D’**, red in **D”**), hub marker Armadillo, as well as spectrosome/fusome marker α spectrin (**B**, red in **B’**) in *Tj>E(Pc) shRNA* testes. (**B-B’**) Over-proliferating germ cells within one cyst (yellow dashed line based on Armadillo signal) had both round spectrosome (yellow arrowhead) and branched fusome (yellow arrow). Scale bar: 20μm.
(TIF)

S4 Fig Overpopulated germ cells in *Tj>E(Pc) RNAi* testes at transit-amplifying stage were Bam-positive. (**A-A’**) In *Bam-HA*, *Tj-Gal4* control testes, immunostaining with anti-HA (red) and anti-Vasa (green) showed Bam expression in 4- to 16- spermatogonial cells (red dashed line). In *Bam-HA*, *Tj>E(Pc) dsRNA* testes (**B-B’**) and *Bam-HA*, *Tj>E(Pc) shRNA* testes (**C-C’**): Bam was detectable in spermatogonial tumor cells (red dashed line labeled over-proliferative cell zone and yellow dashed line labeled individual spermatogonial tumor cysts). Asterisk: hub. Scale bar: 20μm.
(TIF)

S5 Fig Germline tumor cells in *nos>upd* or *bam*^*1*^*/bam*^*114*^ testes were not positively stained with anti-Zfh-1. (**A-A”**) In *nos>upd* testes, Vasa-positive GSC-like cells (**A**, green in **A”**) were intermingled with Zfh-1-positive cells (**A’**, red in **A”**). Scale bar: 20μm. White dashed region enlarged in **B-B”**. Vasa-positive cells (yellow arrowheads in **B**, **B”**) were not stained with antibodies against Zfh-1 (yellow arrowhead in **B’**, **B”**). Scale bar: 10μm. (**C-C”**) In *bam*^*1*^*/bam*^*114*^ testes, spermatogonial tumor cells (white dashed circle) were not stained with antibodies against Zfh-1. Scale bar: 50μm. (**D-D”**) Enlarged apical tip (white dashed square in **C-C”**): Zfh-1 only detectable at the apical tip (arrowhead in **D-D”**). Scale bar: 20μm.
(TIF)

S6 Fig Reducing E(z) significantly enhanced the tumor phenotype in *Tj>E(Pc) shRNA* testes. (**A-C**) In *Tj>E(Pc) shRNA* testes, *E(Pc)* knockdown in cyst cells led to both somatic and germline tumor shown as expansion of DAPI bright region (white dashed line). Scale bar: 100μm. (**D**) Quantification of the penetrance and severity of the tumor phenotype at different genetic backgrounds. Testes were dissected from flies 5 days after shifting to 29°C. **P<0.01, ****P<0.0001, chi-square test.
(TIF)

S7 Fig Knockdown of *E(Pc)* in hub cells did not lead to any detectable defect. (**A-A’”**) In *upd-Gal4* control testes, transit-amplifying stage germ cells (yellow dashed line) with DAPI bright nuclei localize at the apical tip of testis. (**B-B’”**) In *upd>E(Pc) dsRNA* testes, no expansion of DAPI bright region was observed as in *Tj>E(Pc) RNAi* testes. Refer to Fig 2. White outline: hub region. Scale bar: 20μm.
(TIF)

S8 Fig *E(Pc)* mutant cyst cell clones induced ectopic Zfh-1 expression. (**A-B”’**) 5D After clonal induction (ACI), GFP labeled wild-type CySCs (yellow arrowhead) were Zfh-1 positive, while GFP positive cyst cells (yellow arrows) had none (**A”**) or diminished Zfh-1 expression (**B”**). (**C-C”’**) 5D ACI, Zfh-1 was still detectable in GFP-labeled Eya-positive *E(Pc)* mutant cyst cells (yellow arrows). Asterisk: hub. Scale bar: 10μm. (**D-D’**) GFP positive CySCs localized at the apical tip DAPI bright region. In the same testes (**E-E’**), extra DAPI bright cells (yellow dashed line), including Zfh-1-positive *E(Pc)* mutant cyst cells (yellow arrow in D), were detected. Asterisk: hub. Scale bar: 20μm.
(TIF)

S9 Fig *E(Pc)* shRNA knockdown reduced GFP signal of the E(Pc) cDNA-GFP transgene. (**A-B**) *Tj>E(Pc) cDNA-GFP* and *Tj>E(Pc) cDNA-GFP*, *E(Pc) shRNA* testes were mounted on the same slide for comparing the GFP signal. Asterisk: hub. Scale bar: 20μm. (**C**) Quantification of the GFP intensity. *Tj>E(Pc) cDNA-GFP*: 33141 ± 6499 (Mean ± SD, N = 22); *Tj>E(Pc) cDNA-GFP*, *E(Pc) shRNA*: 11523 ± 3811 (N = 50). **** *P*<0.0001. Two-tailed t test.
(TIF)

S10 Fig Overexpression of E(Pc) in cyst cells cause defects in maintenance of Zfh-1-positive early stage cyst cells (including CySCs) and GSCs. (**A-B**) Immunostaining using Vasa (germ cell marker) and Zfh-1 (early cyst cell marker) in *Tj-Gal4* control testes (**A**) and *Tj>E(Pc) cDNA* testes (**B**). GSCs labeled by white dots and Zfh-1 positive cells by white arrowhead. Asterisk: hub. Scale bar: 20μm. (**C**) Quantification of Zfh-1-positive cells. *Tj-Gal4*: 31.28 ± 6.69 (Mean ± SD, N = 105); *Tj>E(Pc)*: 20.97 ± 5.62 (N = 68). (**D**) Quantification of GSCs. *Tj-Gal4*: 8.31 ± 2.04 (N = 75); *Tj>E(Pc)*: 4.95 ± 1.35 (N = 78). *** *P*<0.001. Two-tailed t test. (**E-F**) Immunostaining using a pan cyst cell marker Tj in *Tj-Gal4* control testes (**E**) and *Tj > E(Pc)* testes (**F**). Asterisk: hub. Scale bar: 20μm. (**G**) Quantification of Tj-positive cells. *Tj-Gal4*: 56.26 ± 9.61 (Mean ± SD, N = 38); *Tj>E(Pc)*: 51.19 ± 9.69 (N = 47). *P<0.05. Two-tailed t test.
(TIF)

S11 Fig E(Pc) acts in synergy with Tip60 in cyst cells to regulate germ cell differentiation. (**A-B**) Quantification of the penetrance and severity of the germline tumor phenotype at different genetic background. ***P<0.001, ****P<0.0001, chi-square test. (**C**) Quantification of Zfh-1-positive cells. *Tj-Gal4* control testes: 40±6.96 (N = 35), *Tj>Tip60 cDNA* testes: 30.42±8.24 (N = 50). ****P<0.0001, Two-tailed t test. (**D**) Quantification of GSCs. *Tj-Gal4* control testes: 8.11±1.84 (N = 35), *Tj>Tip60 cDNA* testes: 6.64±2.18 (N = 50). **P<0.01, Two-tailed t test.
(TIF)

S12 Fig Enrichment of histone H4 acetylation at *zfh-1* and *yan* gene loci requires normal E(Pc) function in cyst cells. ChIPed DNA in *Tj-Gal4*, *Tj >E(Pc) shRNA* testes for tetra-acetylated histone H4 (H4 ace) were analyzed by qPCR. Enrichment of H4 ace at *zfh-1* (**A**) and *yan* (**B**) loci were normalized to input as percentage of input. Error bars, s.d. for N = 2 biological replicates. *P* value: two-tailed t test.
(TIF)

S1 Table Overlapping genes between ChIP-seq and RNA-seq [upregulated in E(Pc) knockdown testes] data sets. (XLSX)

S2 Table Overlap genes between ChIP-seq and RNA-seq [downregulated in E(Pc) knockdown testes] data sets. (XLSX)

S3 Table Ontology analysis of overlap genes between ChIP-seq and RNA-seq [upregulated in E(Pc) knockdown testes] data sets. (XLSX)

S4 Table Primers used for ChIP-qPCR analyses. (XLSX)
