# Supplementary figures and images for: Enhancer of polycomb coordinates multiple signaling pathways to promote both cyst and germline stem cell differentiation in the Drosophila adult testis
Source: PLoS Genet. 2017 Feb 14;13(2):e1006571. doi: 10.1371/journal.pgen.1006571 (PMC5308785; doi:10.1371/journal.pgen.1006571)

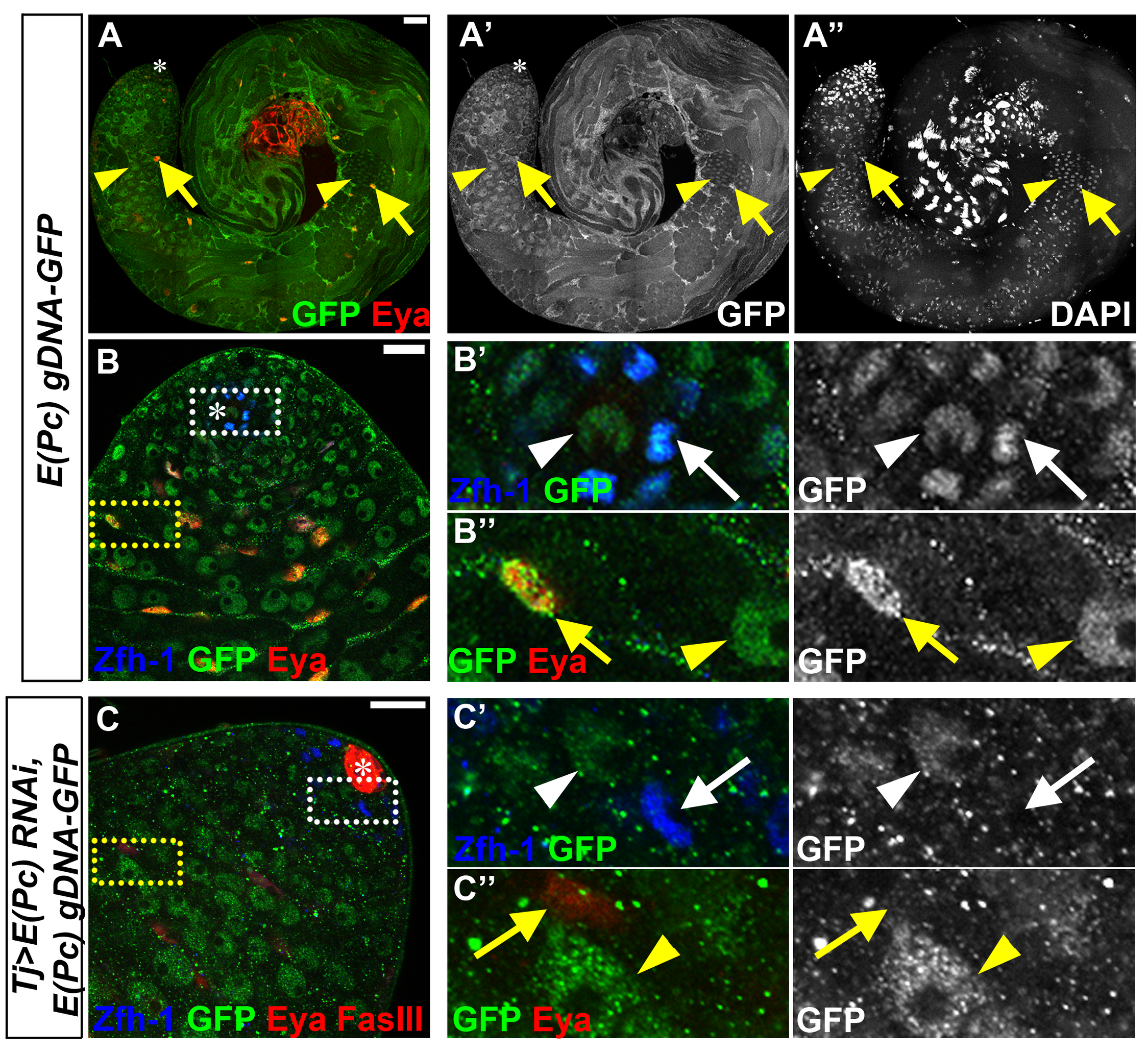

Supplement: S1 Fig — (A-A”) GFP signal in the nuclei of both germ cells (yellow arrowheads) and Eya-positive late-stage cyst cells (yellow arrows). Scale bar: 50μm. (B-B”) At the apical tip, GFP signal in the nuclei of germ cells (white arrowhead in B’ and yellow arrowhead in B”), early cyst cells (Zfh-1-positive cell, labeled by white arrow in B’) and later cyst cells (Eya-positive cell, labeled by yellow arrow in B”). (C-C”) In Tj>E(Pc) RNAi, E(Pc) gDNA-GFP testes, undetectable GFP signal in early cyst cells (Zfh-1-positive cell, labeled by white arrow in C’) and later cyst cells (Eya-positive cell, labeled by yellow arrow in C”), but detectable in germ cells (white arrowhead in C’ and yellow arrowhead in C”). Asterisk: hub. Scale bar: 20μm. (TIF) [file pgen.1006571.s001.tif]

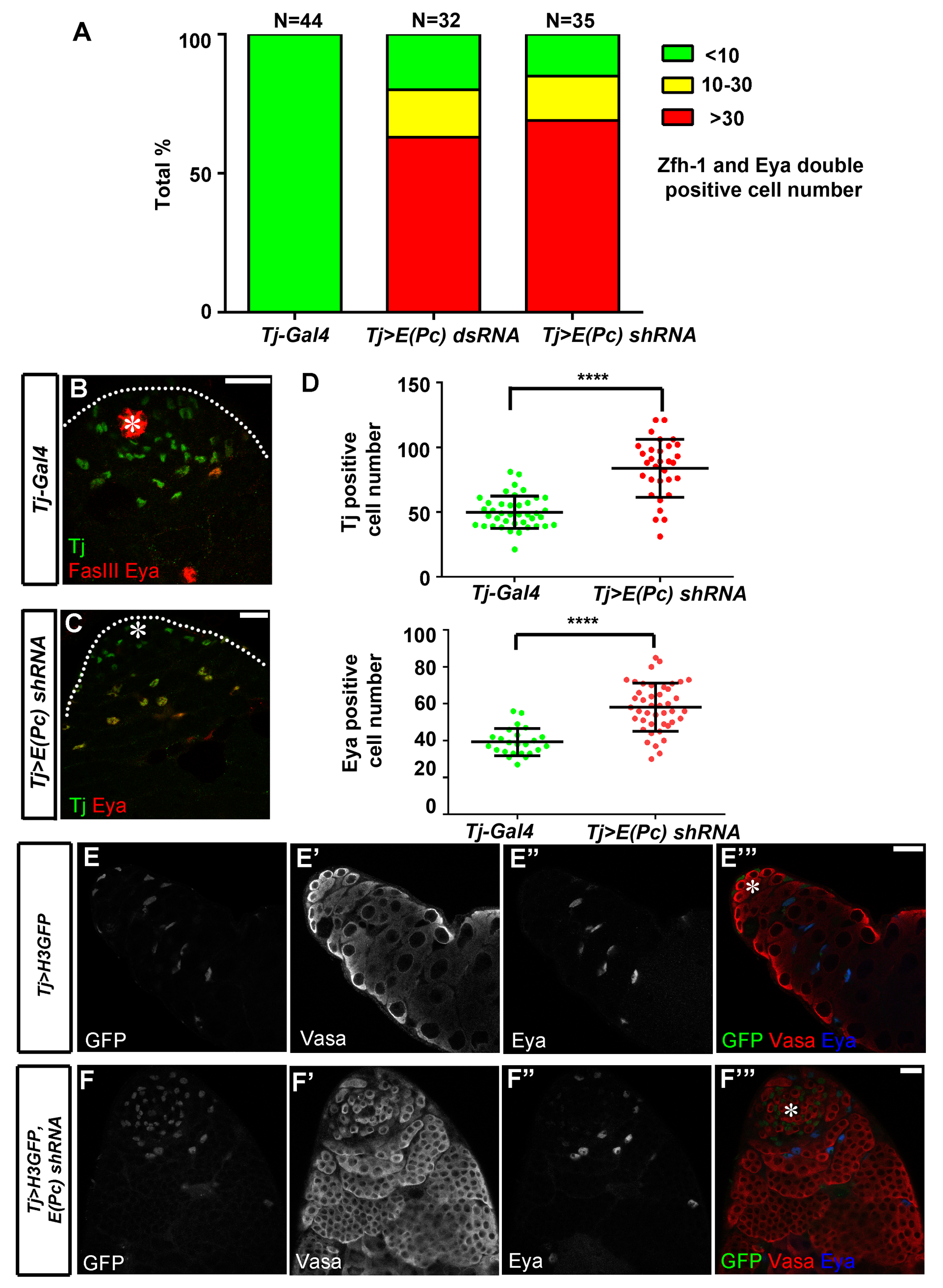

Supplement: S2 Fig — (A) Percentage of testes with <10, 10–30 and >30 Zfh-1- and Eya-double positive cyst cells in different genotyped testes. (B-C) Immunostaining with anti-Tj and Eya in Tj-Gal4 and Tj>E(Pc) shRNA testes. (D) Quantification of Tj-positive cells in Tj-Gal4 control testes: 50 ± 12.49 (Mean ± SD, N = 40) and in Tj>E(Pc) shRNA testes: 83.91 ± 22.41 (N = 31). Quantification of Eya-positive cells at the tip of Tj-Gal4 control testes: 39 ± 7.35 (Mean ± SD, N = 24) and Tj>E(Pc) shRNA testes: 58 ± 13.04 (N = 43). **** P<0.0001, two-tailed t test. (E-F’”) Immunostaining using the germ cell marker Vasa (E’, F’) and a late cyst cell marker Eya (E”, F”) in Tj>H3 GFP and Tj> H3 GFP, E(Pc) shRNA testes. Asterisk: hub. Scale bar: 20μm. (TIF) [file pgen.1006571.s002.tif]

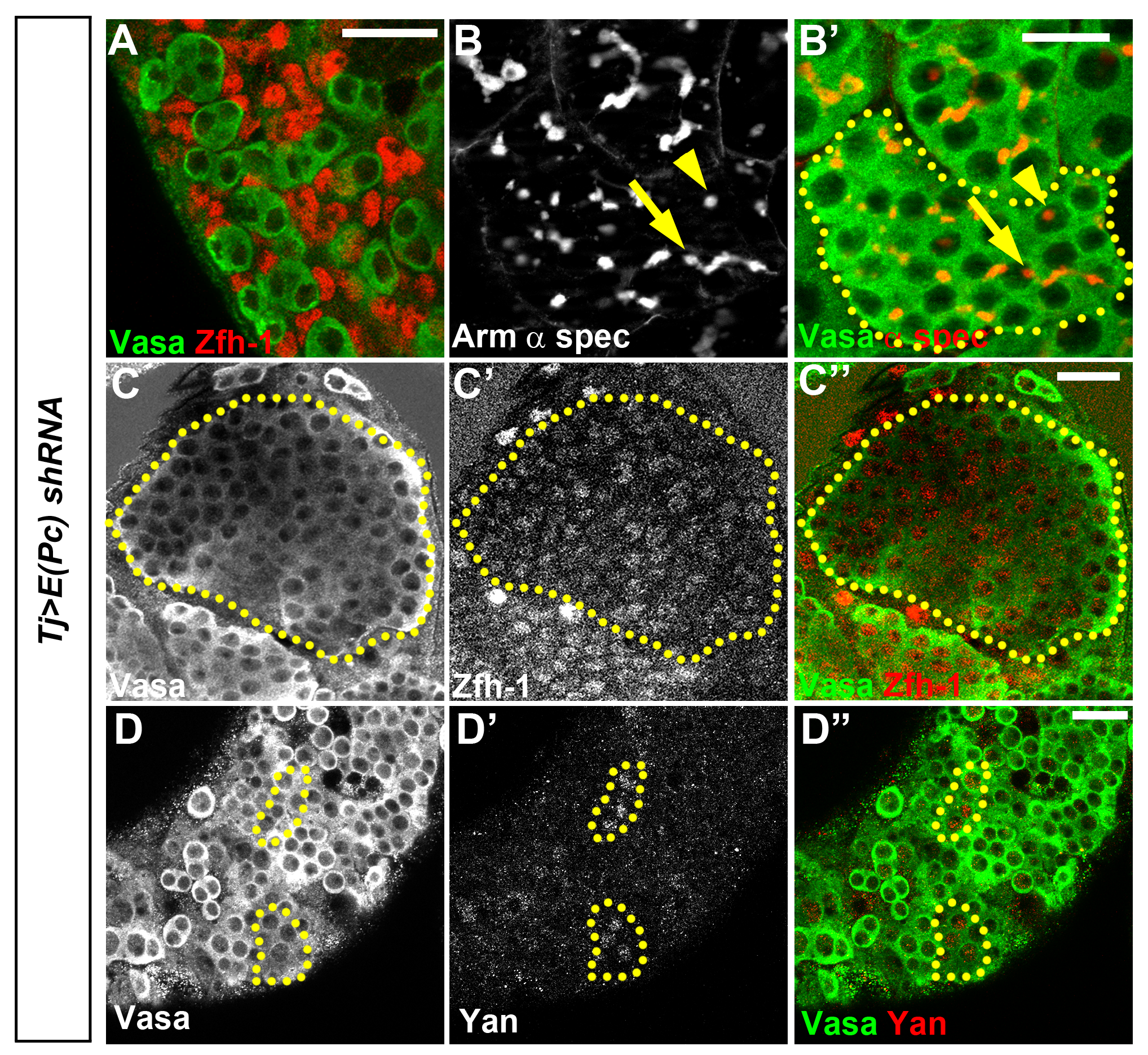

Supplement: S3 Fig — Immunostaining using the germ cell marker Vasa (C and D, green in A, B’, D”), early cyst cell markers Zfh-1 (C’, red in A, C”) and Yan (D’, red in D”), hub marker Armadillo, as well as spectrosome/fusome marker α spectrin (B, red in B’) in Tj>E(Pc) shRNA testes. (B-B’) Over-proliferating germ cells within one cyst (yellow dashed line based on Armadillo signal) had both round spectrosome (yellow arrowhead) and branched fusome (yellow arrow). Scale bar: 20μm. (TIF) [file pgen.1006571.s003.tif]

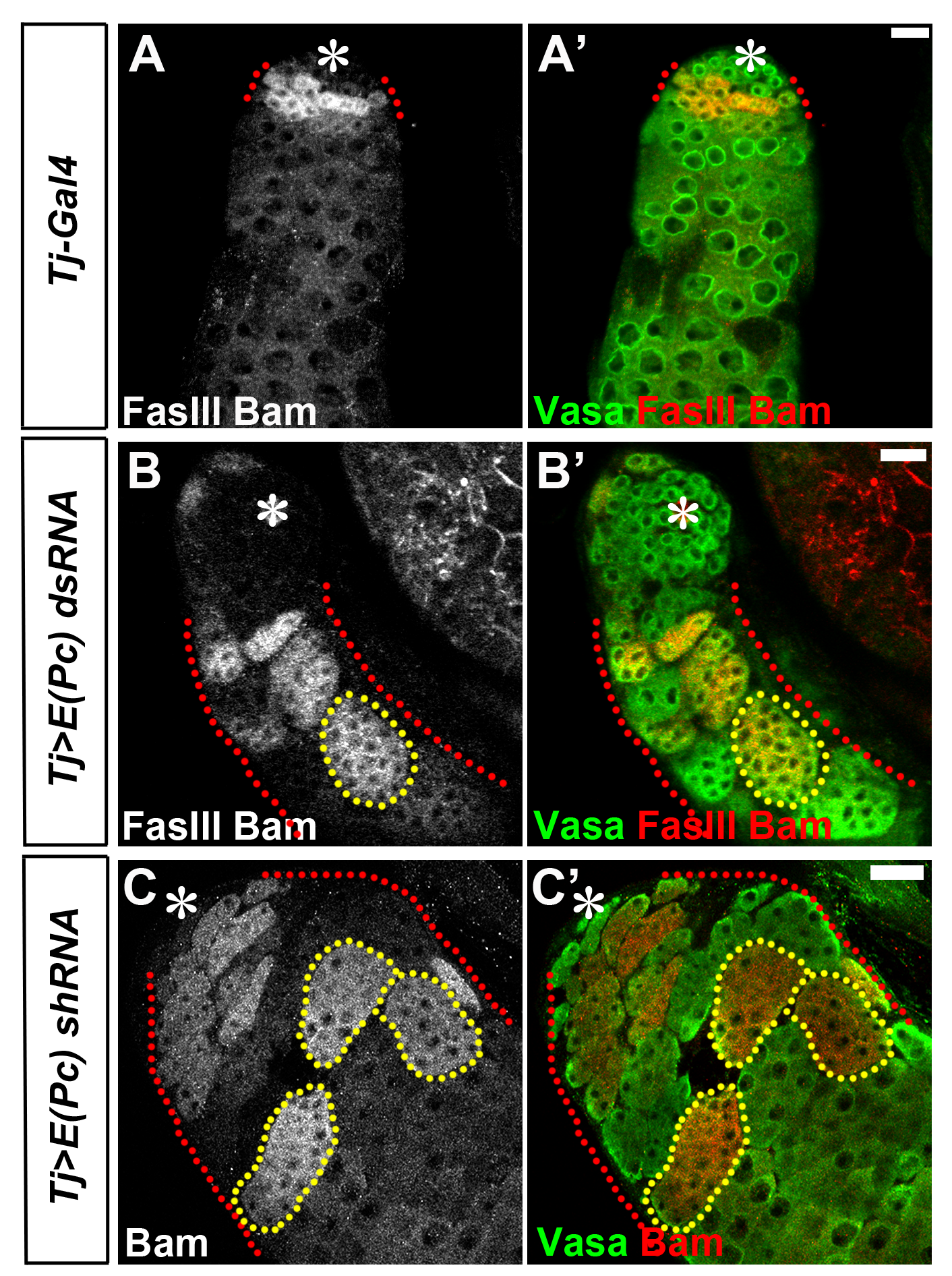

Supplement: S4 Fig — (A-A’) In Bam-HA, Tj-Gal4 control testes, immunostaining with anti-HA (red) and anti-Vasa (green) showed Bam expression in 4- to 16- spermatogonial cells (red dashed line). In Bam-HA, Tj>E(Pc) dsRNA testes (B-B’) and Bam-HA, Tj>E(Pc) shRNA testes (C-C’): Bam was detectable in spermatogonial tumor cells (red dashed line labeled over-proliferative cell zone and yellow dashed line labeled individual spermatogonial tumor cysts). Asterisk: hub. Scale bar: 20μm. (TIF) [file pgen.1006571.s004.tif]

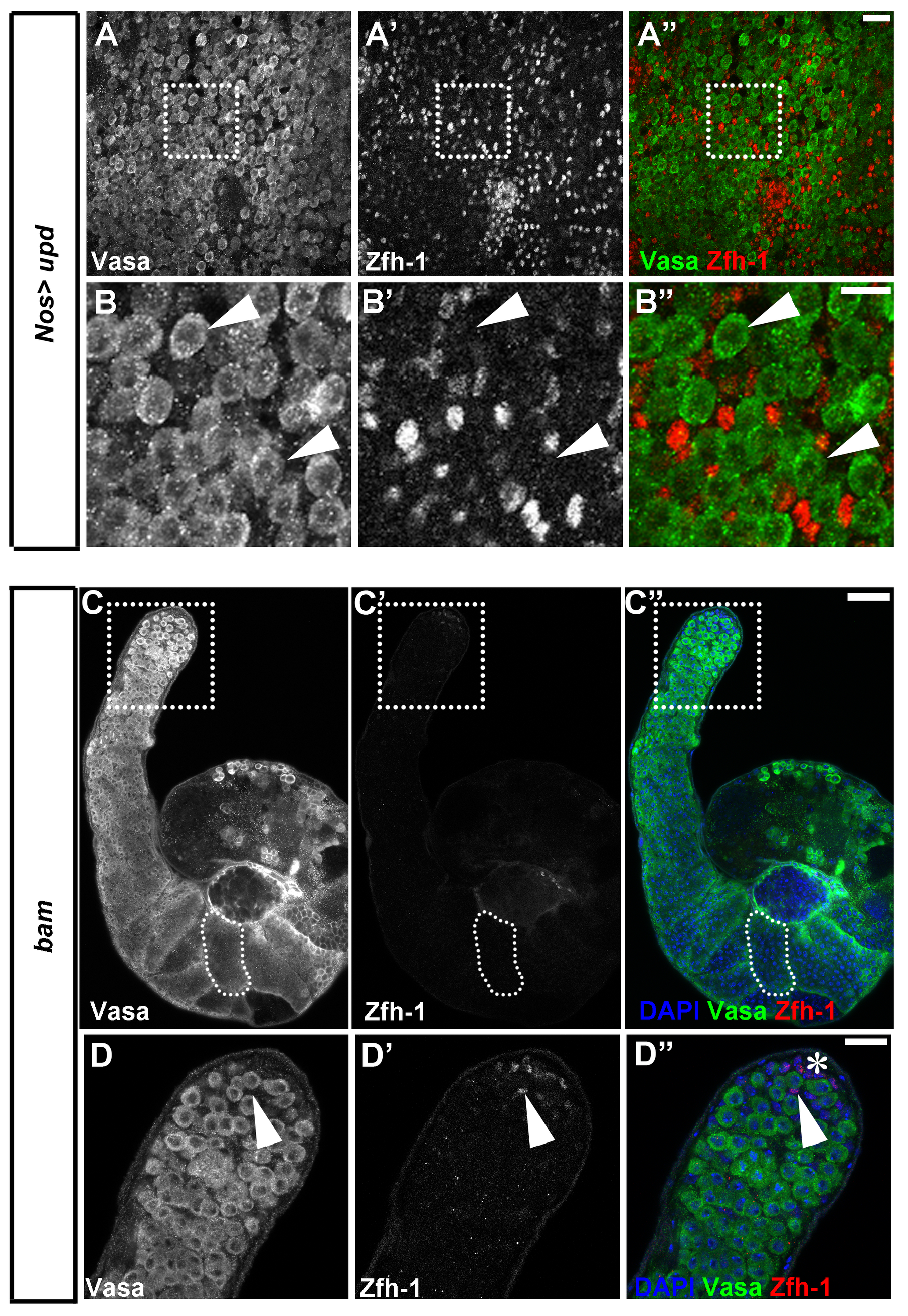

Supplement: S5 Fig — (A-A”) In nos>upd testes, Vasa-positive GSC-like cells (A, green in A”) were intermingled with Zfh-1-positive cells (A’, red in A”). Scale bar: 20μm. White dashed region enlarged in B-B”. Vasa-positive cells (yellow arrowheads in B, B”) were not stained with antibodies against Zfh-1 (yellow arrowhead in B’, B”). Scale bar: 10μm. (C-C”) In bam1/bam114 testes, spermatogonial tumor cells (white dashed circle) were not stained with antibodies against Zfh-1. Scale bar: 50μm. (D-D”) Enlarged apical tip (white dashed square in C-C”): Zfh-1 only detectable at the apical tip (arrowhead in D-D”). Scale bar: 20μm. (TIF) [file pgen.1006571.s005.tif]

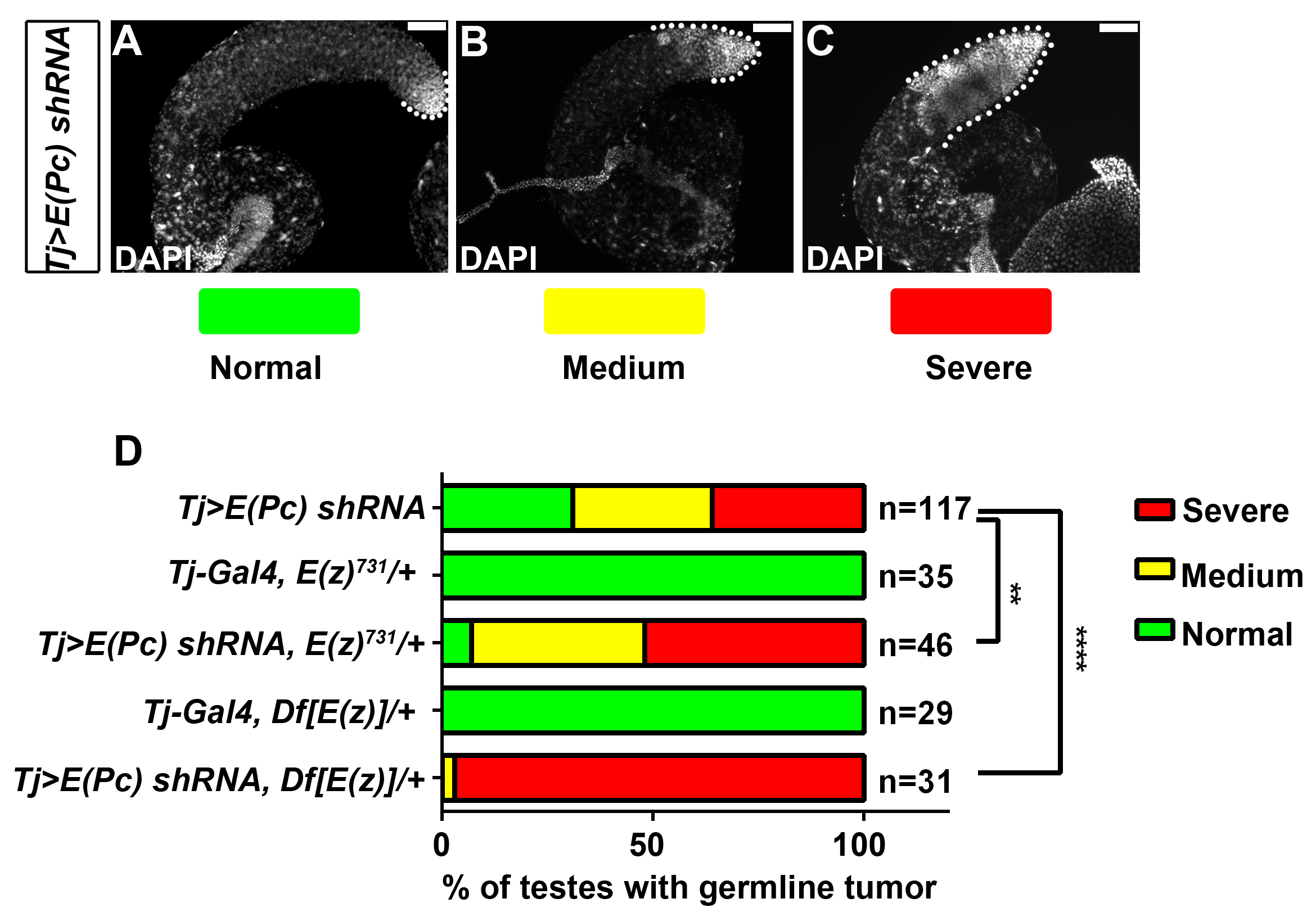

Supplement: S6 Fig — (A-C) In Tj>E(Pc) shRNA testes, E(Pc) knockdown in cyst cells led to both somatic and germline tumor shown as expansion of DAPI bright region (white dashed line). Scale bar: 100μm. (D) Quantification of the penetrance and severity of the tumor phenotype at different genetic backgrounds. Testes were dissected from flies 5 days after shifting to 29°C. **P<0.01, ****P<0.0001, chi-square test. (TIF) [file pgen.1006571.s006.tif]

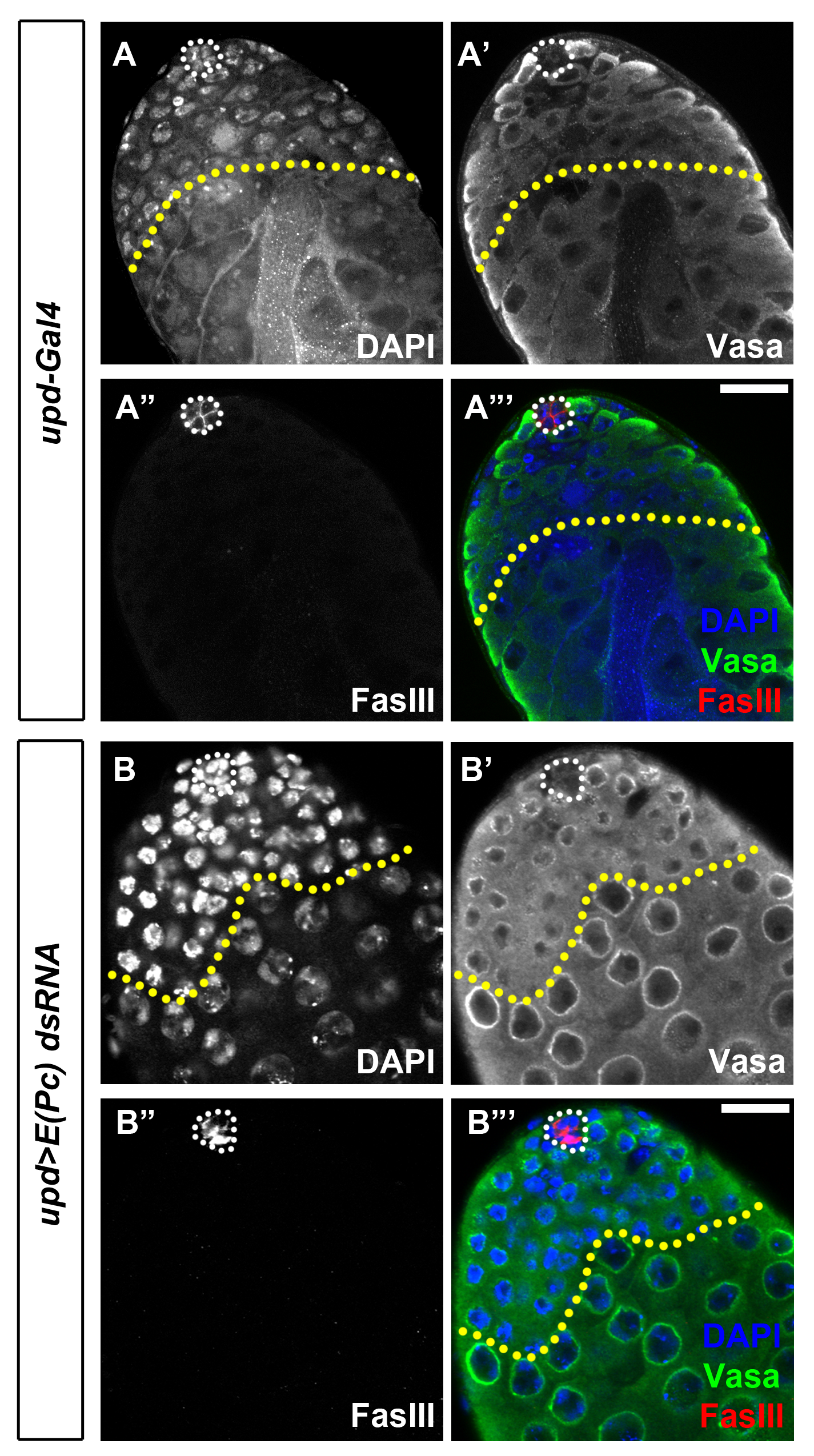

Supplement: S7 Fig — (A-A’”) In upd-Gal4 control testes, transit-amplifying stage germ cells (yellow dashed line) with DAPI bright nuclei localize at the apical tip of testis. (B-B’”) In upd>E(Pc) dsRNA testes, no expansion of DAPI bright region was observed as in Tj>E(Pc) RNAi testes. Refer to Fig 2. White outline: hub region. Scale bar: 20μm. (TIF) [file pgen.1006571.s007.tif]

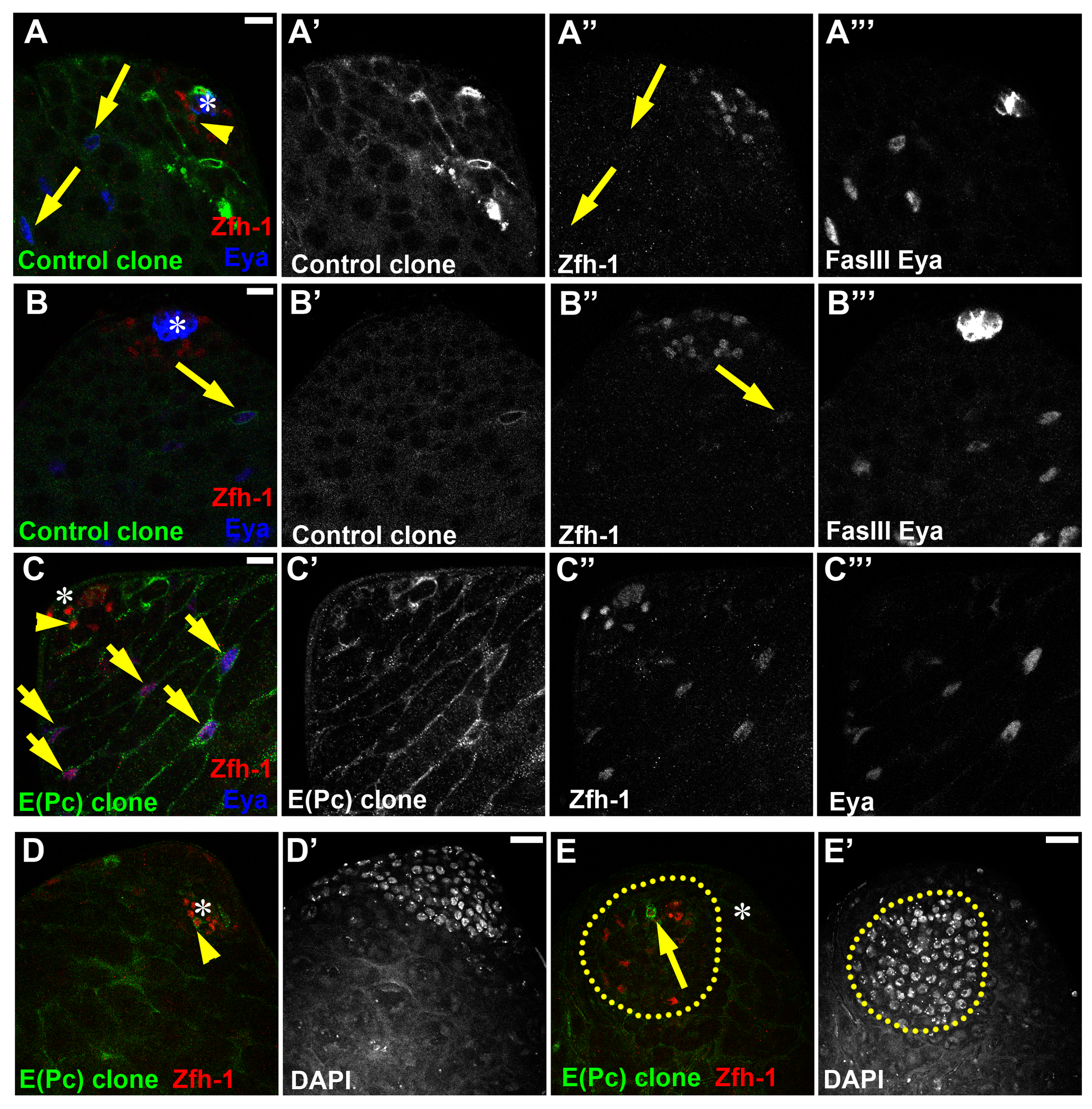

Supplement: S8 Fig — (A-B”’) 5D After clonal induction (ACI), GFP labeled wild-type CySCs (yellow arrowhead) were Zfh-1 positive, while GFP positive cyst cells (yellow arrows) had none (A”) or diminished Zfh-1 expression (B”). (C-C”’) 5D ACI, Zfh-1 was still detectable in GFP-labeled Eya-positive E(Pc) mutant cyst cells (yellow arrows). Asterisk: hub. Scale bar: 10μm. (D-D’) GFP positive CySCs localized at the apical tip DAPI bright region. In the same testes (E-E’), extra DAPI bright cells (yellow dashed line), including Zfh-1-positive E(Pc) mutant cyst cells (yellow arrow in D), were detected. Asterisk: hub. Scale bar: 20μm. (TIF) [file pgen.1006571.s008.tif]

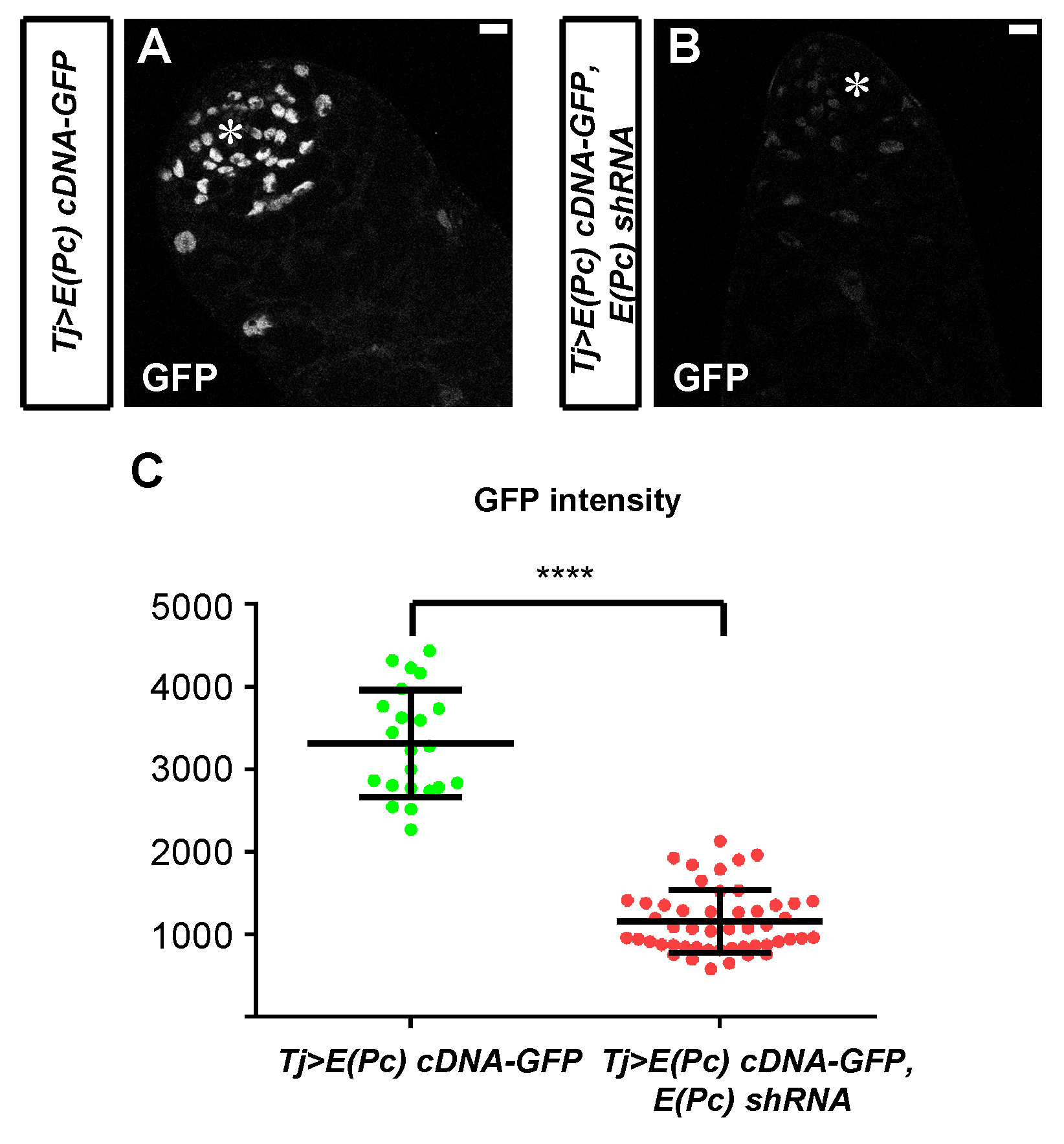

Supplement: S9 Fig — (A-B) Tj>E(Pc) cDNA-GFP and Tj>E(Pc) cDNA-GFP, E(Pc) shRNA testes were mounted on the same slide for comparing the GFP signal. Asterisk: hub. Scale bar: 20μm. (C) Quantification of the GFP intensity. Tj>E(Pc) cDNA-GFP: 33141 ± 6499 (Mean ± SD, N = 22); Tj>E(Pc) cDNA-GFP, E(Pc) shRNA: 11523 ± 3811 (N = 50). **** P<0.0001. Two-tailed t test. (TIF) [file pgen.1006571.s009.tif]

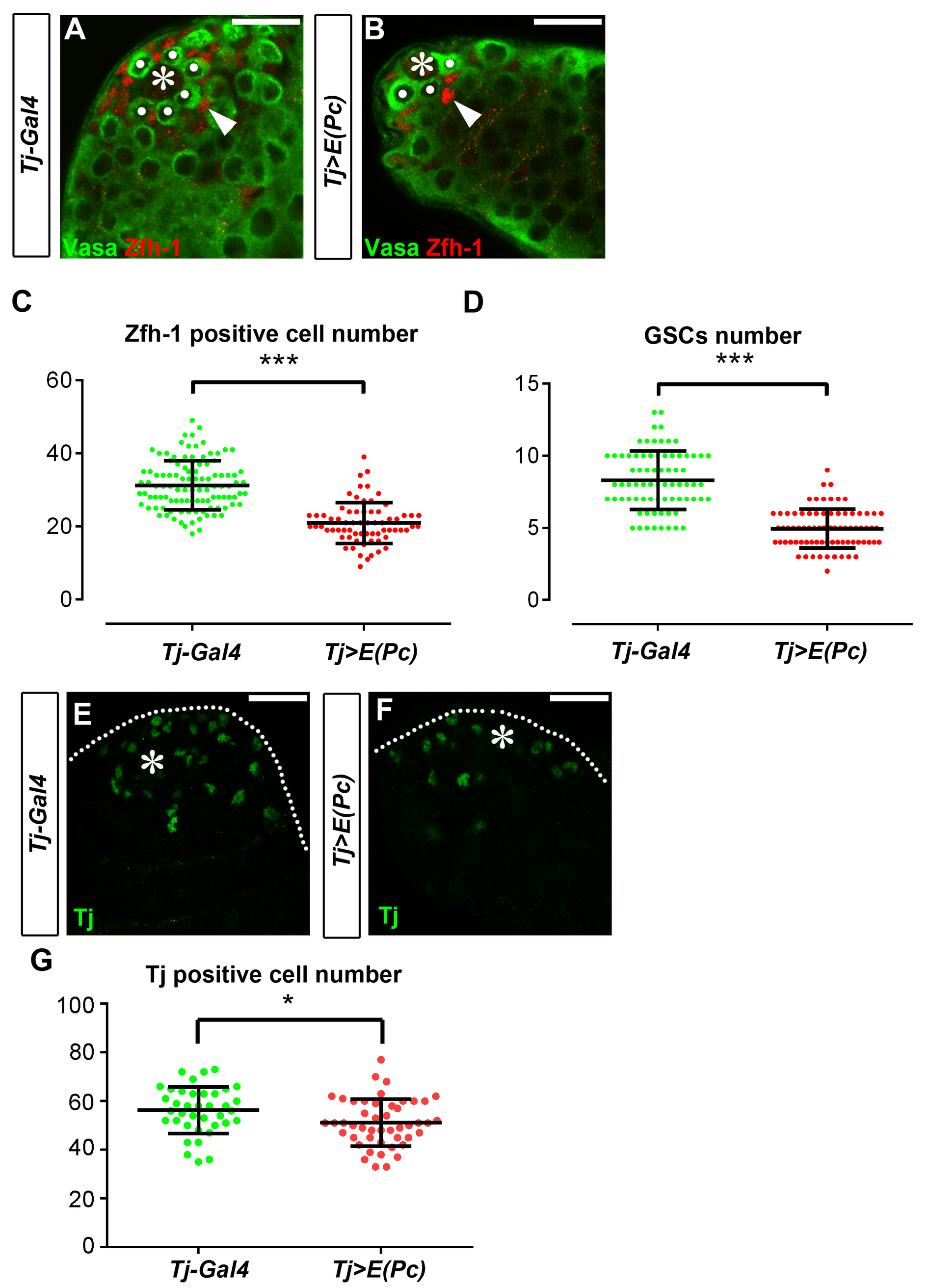

Supplement: S10 Fig — (A-B) Immunostaining using Vasa (germ cell marker) and Zfh-1 (early cyst cell marker) in Tj-Gal4 control testes (A) and Tj>E(Pc) cDNA testes (B). GSCs labeled by white dots and Zfh-1 positive cells by white arrowhead. Asterisk: hub. Scale bar: 20μm. (C) Quantification of Zfh-1-positive cells. Tj-Gal4: 31.28 ± 6.69 (Mean ± SD, N = 105); Tj>E(Pc): 20.97 ± 5.62 (N = 68). (D) Quantification of GSCs. Tj-Gal4: 8.31 ± 2.04 (N = 75); Tj>E(Pc): 4.95 ± 1.35 (N = 78). *** P<0.001. Two-tailed t test. (E-F) Immunostaining using a pan cyst cell marker Tj in Tj-Gal4 control testes (E) and Tj > E(Pc) testes (F). Asterisk: hub. Scale bar: 20μm. (G) Quantification of Tj-positive cells. Tj-Gal4: 56.26 ± 9.61 (Mean ± SD, N = 38); Tj>E(Pc): 51.19 ± 9.69 (N = 47). *P<0.05. Two-tailed t test. (TIF) [file pgen.1006571.s010.tif]

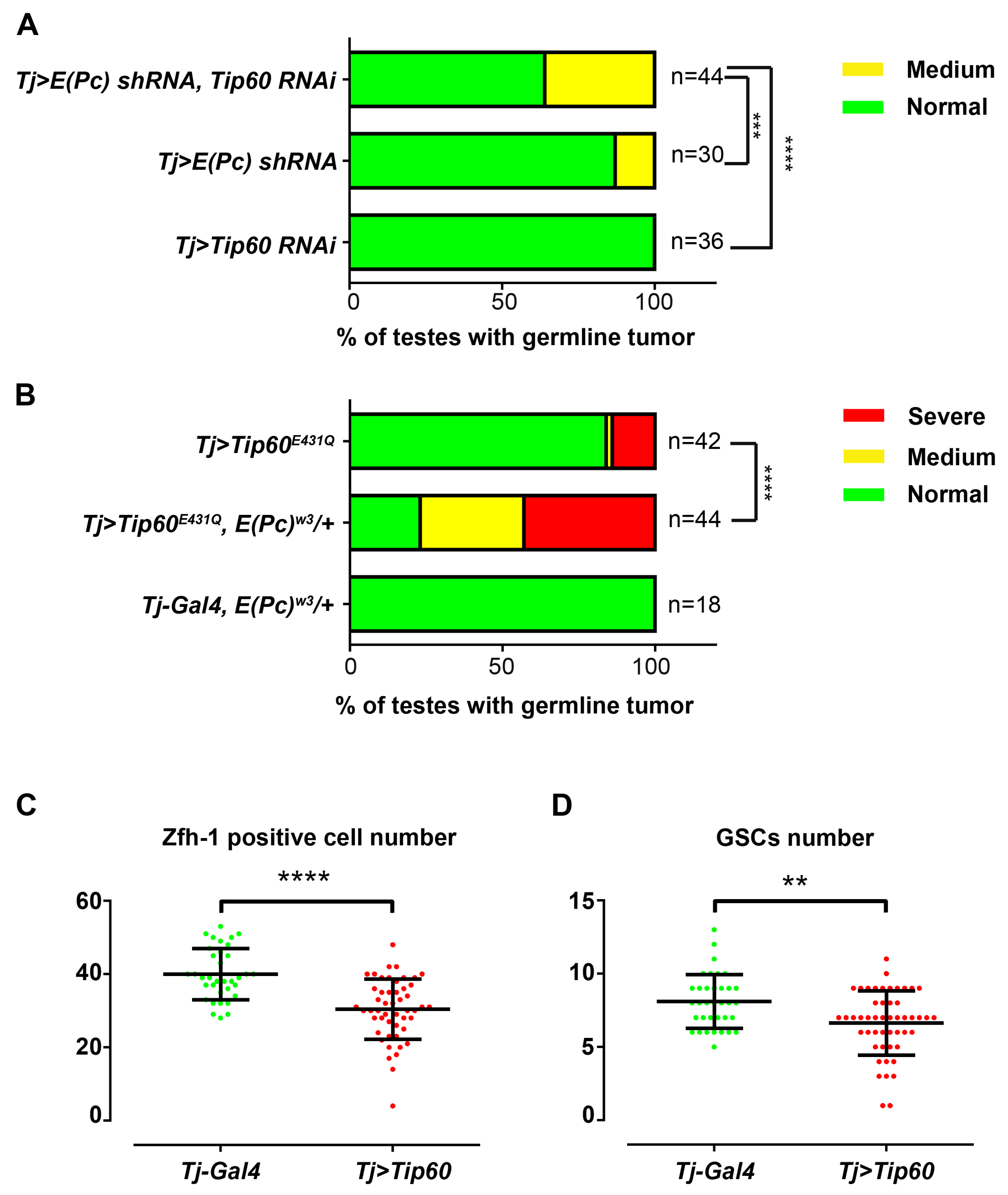

Supplement: S11 Fig — (A-B) Quantification of the penetrance and severity of the germline tumor phenotype at different genetic background. ***P<0.001, ****P<0.0001, chi-square test. (C) Quantification of Zfh-1-positive cells. Tj-Gal4 control testes: 40±6.96 (N = 35), Tj>Tip60 cDNA testes: 30.42±8.24 (N = 50). ****P<0.0001, Two-tailed t test. (D) Quantification of GSCs. Tj-Gal4 control testes: 8.11±1.84 (N = 35), Tj>Tip60 cDNA testes: 6.64±2.18 (N = 50). **P<0.01, Two-tailed t test. (TIF) [file pgen.1006571.s011.tif]

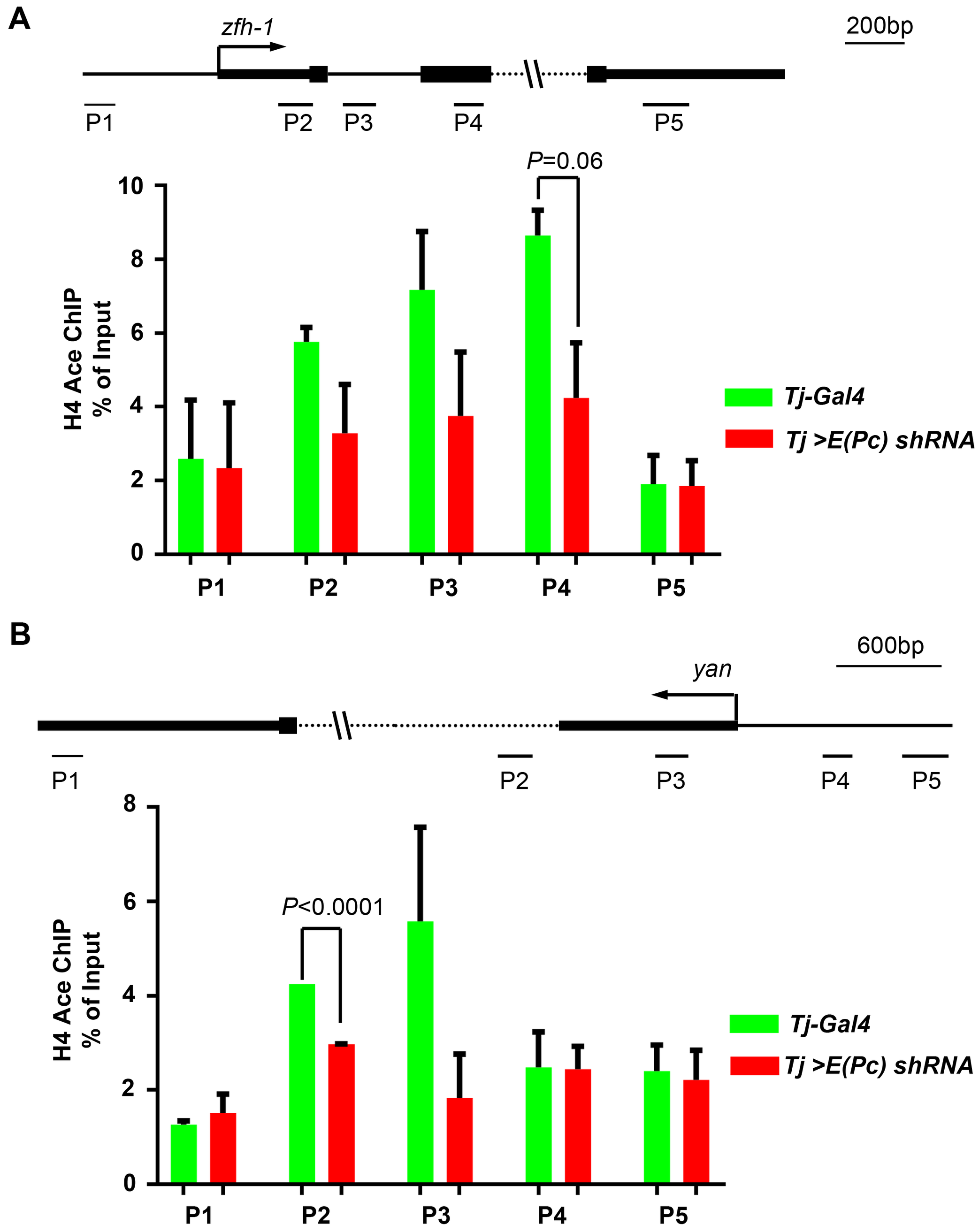

Supplement: S12 Fig — ChIPed DNA in Tj-Gal4, Tj >E(Pc) shRNA testes for tetra-acetylated histone H4 (H4 ace) were analyzed by qPCR. Enrichment of H4 ace at zfh-1 (A) and yan (B) loci were normalized to input as percentage of input. Error bars, s.d. for N = 2 biological replicates. P value: two-tailed t test. (TIF) [file pgen.1006571.s012.tif]
